# Detecting Spontaneous Neural Oscillation Events in Primate Auditory Cortex

**DOI:** 10.1523/ENEURO.0281-21.2022

**Published:** 2022-08-18

**Authors:** Samuel A. Neymotin, Idan Tal, Annamaria Barczak, Monica N. O’Connell, Tammy McGinnis, Noah Markowitz, Elizabeth Espinal, Erica Griffith, Haroon Anwar, Salvador Dura-Bernal, Charles E. Schroeder, William W. Lytton, Stephanie R. Jones, Stephan Bickel, Peter Lakatos

**Affiliations:** 1 Center for Biomedical Imaging and Neuromodulation, Nathan Kline Institute for Psychiatric Research, Orangeburg, NY 10962; 2 Department Psychiatry, New York University Grossman School of Medicine, New York, NY 10016; 3 Departments of Neurosurgery and Psychiatry, Columbia University College of Physicians and Surgeons, New York, NY 10032; 4 Department Neurology and Neurosurgery, The Feinstein Institutes for Medical Research at Northwell Health, Manhasset, NY 11030; 5 Department Physiology and Pharmacology, State University of New York Downstate Medical Center, Brooklyn, NY 11203; 6 Department Neurology, Kings County Hospital Center, Brooklyn, NY 11203; 7 Department Neuroscience and Carney Institute for Brain Science, Brown University, Providence, RI 02906

**Keywords:** auditory cortex, current-source density, electrophysiology, local field potential, oscillations, rhythms

## Abstract

Electrophysiological oscillations in the brain have been shown to occur as multicycle events, with onset and offset dependent on behavioral and cognitive state. To provide a baseline for state-related and task-related events, we quantified oscillation features in resting-state recordings. We developed an open-source wavelet-based tool to detect and characterize such oscillation events (OEvents) and exemplify the use of this tool in both simulations and two invasively-recorded electrophysiology datasets: one from human, and one from nonhuman primate (NHP) auditory system. After removing incidentally occurring event-related potentials (ERPs), we used OEvents to quantify oscillation features. We identified ∼2 million oscillation events, classified within traditional frequency bands: δ, θ, α, β, low γ, γ, and high γ. Oscillation events of 1–44 cycles could be identified in at least one frequency band 90% of the time in human and NHP recordings. Individual oscillation events were characterized by nonconstant frequency and amplitude. This result necessarily contrasts with prior studies which assumed frequency constancy, but is consistent with evidence from event-associated oscillations. We measured oscillation event duration, frequency span, and waveform shape. Oscillations tended to exhibit multiple cycles per event, verifiable by comparing filtered to unfiltered waveforms. In addition to the clear intraevent rhythmicity, there was also evidence of interevent rhythmicity within bands, demonstrated by finding that coefficient of variation of interval distributions and Fano factor (FF) measures differed significantly from a Poisson distribution assumption. Overall, our study provides an easy-to-use tool to study oscillation events at the single-trial level or in ongoing recordings, and demonstrates that rhythmic, multicycle oscillation events dominate auditory cortical dynamics.

## Significance Statement

To provide a baseline for auditory system cortical dynamics, we quantified neuronal oscillation event features in resting-state recordings of the auditory system. We found that even at rest, event-like oscillations are the dominant operational mode of the auditory cortex in both humans and nonhuman primates (NHPs). Our results highlight the importance of the auditory system’s rhythmic neuronal fluctuations in setting the context on top of which auditory processing necessary for behavior and cognition occurs. In addition, we demonstrate the importance of studying basic features of oscillation events in ongoing and single-trial recordings to understand their role in cognition and the mechanisms generating them.

## Introduction

Oscillation classification remains understudied because of lack of common criteria, but is critical for understanding the auditory system, where oscillations in air pressure because of sound are reflected by oscillations in cortex at multiple time scales (Rosen, 1992). Auditory cortex oscillation modulation is dependent on arousal, with evidence of correlations of amplitude and attention (Lakatos et al., 2004), as well as distinct timing changes with musical rhythms (Fujioka et al., 2009). Oscillation-perception correlations have also been proposed for other sensory modalities and for motor coordination (Gray et al., 1989).

Intrinsic cortical oscillations consist of rhythmic and brief, pulse-like neuronal activity patterns, which co-occur in electrophysiological recordings (Jones, 2016; Obleser et al., 2017; Tal et al., 2020). These patterns manifest differently across frequency bands and brain regions during various task-dependent brain states (Buzsáki and Draguhn, 2004). Neural oscillations have functional correlates, with ongoing events providing a background context on top of which behaviorally and cognitively relevant information is transmitted (Lakatos et al., 2008). Oscillations form dynamically-changing functional networks based on their matched or mismatched phases (Fries, 2015; Barczak et al., 2018). Intrinsic oscillations are independent of stereotypical event-related potentials (ERPs), which may be prolonged enough to produce a single cycle of oscillation.

δ (0.5–3 Hz), θ (4–8 Hz), α (9–14 Hz), β (15–30 Hz), γ (30–100 Hz), and higher frequency oscillations occur as multicycle events, with onset and offset dependent on behavioral and cognitive state (Buzsáki, 2002; Lakatos et al., 2008). A prominent example is the primate α rhythm (9–14 Hz) observed in the visual cortex (Lopes Da Silva and Storm Van Leeuwen, 1977). Rhythms in the same frequency range have been observed in auditory, somatosensory, and motor cortices and subcortical structures (Haegens et al., 2015; Lakatos et al., 2016; Halgren et al., 2019).

The presence of high spectral power in a neural signal does not necessarily indicate an intrinsic oscillation. This is true particularly for one-cycle or two-cycle events which could arise stochastically (Jones, 2016; Cole and Voytek, 2017; Tal et al., 2020). For example, high-amplitude, single-cycle waveforms with 50 ms duration could be mistaken for 20-Hz oscillations (Sherman et al., 2016). If underlying neural generators are stochastic, high spectral power may reflect temporal domain features from synaptic time constants or other sources (Jones, 2016; Cole and Voytek, 2017). Recent studies demonstrated recurring brief events in high-frequency γ (Lakatos et al., 2004; Lundqvist et al., 2016), and in β (Sherman et al., 2016; Shin et al., 2017; Bonaiuto et al., 2021; Law et al., 2022) ranges. Therefore, care must be taken to distinguish nonoscillation waveforms from clear multicycle oscillations. The relevance of this dichotomy for particular frequency bands, behavioral conditions, and brain regions remains to be determined.

Our aim was to examine nontask-related neuronal activity, recorded over long time scales (minutes) in awake, resting state conditions in humans and nonhuman primates (NHPs), to better understand basic features and temporal properties of physiologically-relevant oscillations in auditory cortex. We used two invasively-recorded electrophysiology datasets: (1) laminar local field potential (LFP) recordings from NHP primary auditory cortex (A1), and (2) intracranial electroencephalogram (iEEG) recordings from human superior temporal gyrus (STG). We extracted moderate to high-power oscillation events from wavelet transform spectrograms, and determined their basic properties. These included temporal and frequency span, peak frequency, peak power, number of cycles, number of local peaks in filtered waveforms, correlation between filtered and raw signal (Filter-match), and rhythmicity across individual events.

Our analysis revealed clear evidence of multicycle oscillation events in all frequency ranges (average: three to four cycles per event), verified by inspecting unfiltered waveforms. Event occurrence in each frequency band displayed rhythmicity, quantified through analysis of interevent intervals (IEIs). Our data and analyses demonstrate several characteristics of oscillation events that may be studied in ongoing or single-trial data. Importantly, we demonstrate that multicycle oscillation events are prominent in resting state auditory cortex dynamics, and that these events recur quasi-rhythmically over time.

## Materials and Methods

### Datasets

We used two datasets of neuronal activity, invasively recorded over longer time scales (minutes) in nontask related conditions: (1) laminar electrode array LFPs from NHP A1 (92 recordings totaling 487.8 min); (2) iEEG from human STG (eight recordings totaling 37 min). In all analyses (except for specific examples), the data were pooled across subjects.

**Figure 1.**
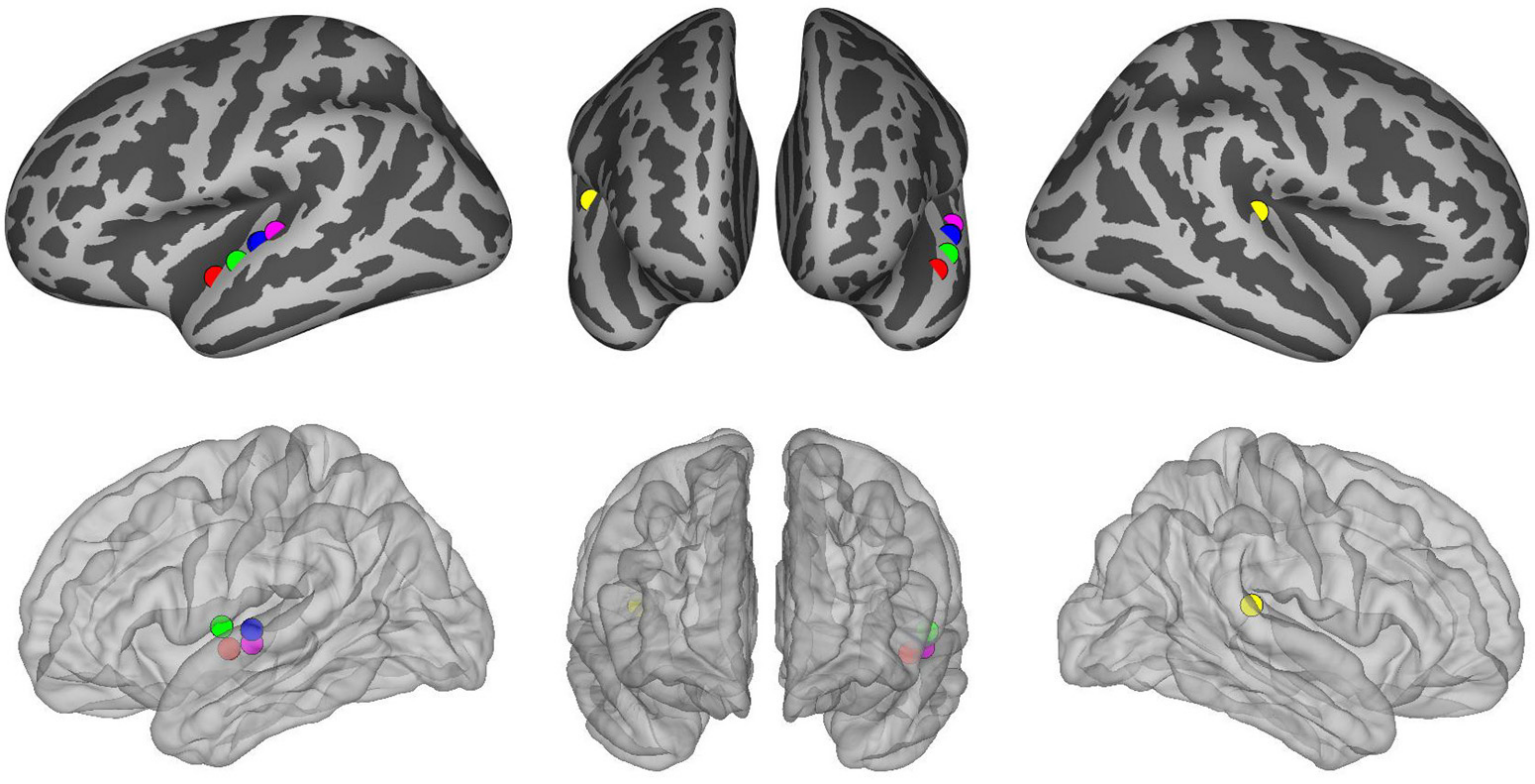
Locations of the iEEG electrodes used for human electrophysiology recordings included in this study, overlayed on a standard average brain. Colors represent different patients.

(1) NHP electrophysiological data were recorded during acute penetrations of area A1 of the auditory cortex of 1 male and three female rhesus macaques weighing 5–8 kg, who had been prepared surgically for chronic awake electrophysiological recordings. Before surgery, each animal was adapted to a custom fitted primate chair and to the recording chamber. All procedures were approved in advance by the Animal Care and Use Committee. Preparation of subjects for chronic awake intracortical recording was performed under general anesthesia using aseptic techniques. To provide access to the brain, either Cilux (Crist Instruments) or polyetheretherketone (PEEK; Rogue Research Inc.) recording chambers were positioned normal to the cortical surface of the superior temporal plane for orthogonal penetration of A1. These recording chambers and either socketed Plexiglas bars or a PEEK headpost (both used to permit painless head restraint) were secured to the skull with ceramic screws and embedded in dental acrylic. Each NHP was given a minimum of six weeks for postoperative recovery before behavioral training and data collection began. Before and after recordings, NHPs had full access to fluids and food. No rewards were offered during recordings. The data were recorded during waking rest with eyes mostly open, while the macaques were in a dark room. During recordings, NHPs were head-fixed and linear array multielectrodes (23 contacts with 100-, 125-, or 150-μm intercontact spacing, Plexon Inc.) were acutely positioned to sample all cortical layers of A1. Neuroelectric signals were continuously recorded with a sampling rate of 44 kHz using the Alpha Omega SnR system. We note that since the monkeys were in a dark room, we would not expect visual inputs to influence the outcome in the auditory cortex. However, we know that eye movements influence oscillatory activity in auditory cortex even in the dark, and based on some recent results from the lab (P. Lakatos, C.E. Schroeder, unpublished data), eyes closed versus eyes open might influence dynamics in the auditory cortex as well.

(2) The iEEG data, previously published (Di Liberto et al., 2020; Shamma et al., 2021), was recorded in medically-intractable epilepsy patients that underwent stereotactic depth electrode placement as part of their epilepsy surgery workup. Electrode placement was based on clinical criteria only (Fig. 1). All patients provided written informed consent monitored by the institutional review board. Electrode localization methods have been described previously (Groppe et al., 2017). The data were recorded using a Tucker Davis Technologies amplifier with a sampling rate of 3000 Hz. The data were recorded during waking rest with eyes closed. While we cannot exclude a possible effect of medications on brain oscillations in general, a systematic effect is unlikely because: (1) these recordings are usually done when patients are either off medications or being tapered off, (2) different patients are administered multiple different medications which makes a systematic effect across patients unlikely, and (3) the patients were not administered barbiturates. Benzodiazepines were only used to abort seizures and we avoided recording experiments following seizures that require its administration. Note that our recording systems store the iEEG data in either V or μV formats, depending on the recording system used.

### Data processing

All analyses from the NHP dataset were run on current-source density (CSD) signals, calculated as the second spatial derivative of laminar LFP recordings. This was done to reduce potential issues related to volume conducted activity. We estimate CSD using laminar LFP recordings. To reduce computation time, we only used channels that included the supragranular, granular, and infragranular current sinks in response to preferred frequency tones, considered “active” since they measure depolarizing transmembrane currents (Lakatos et al., 2016). In humans, it is not as simple to calculate CSD because of more restricted electrode arrangements. Thus, all analyses from the iEEG dataset used the recorded signals themselves without taking any spatial derivative.

### Removal of externally-driven events

**Figure 2.**
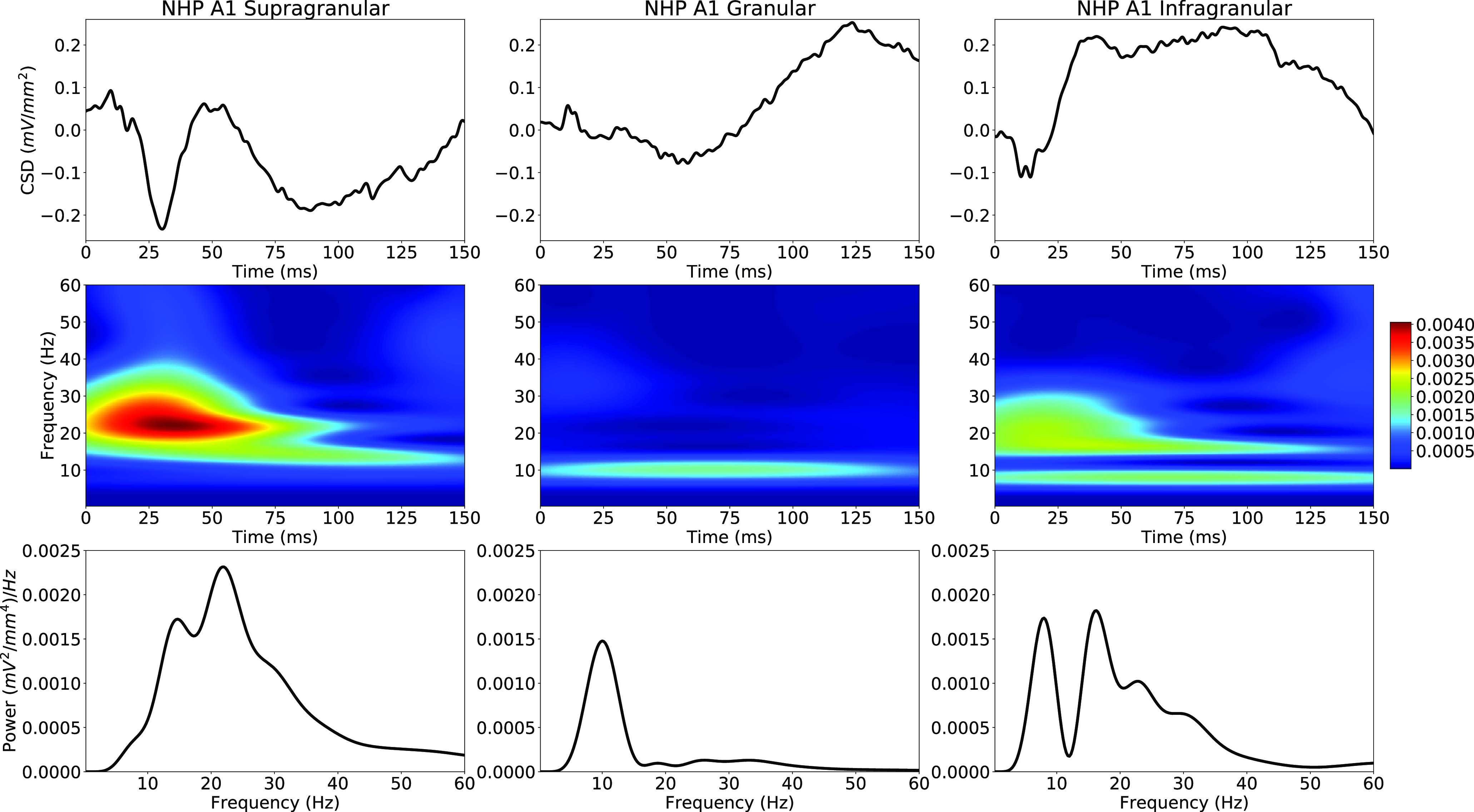
Stereotyped ERPs in NHP A1: supragranular, granular, infragranular layers (left to right; 50-dB clicks in NHP). Top, Average ERP waveforms (click at 0 ms). Middle, Wavelet transform spectrograms (values are in units of Power; note that these spectrograms are not normalized). Bottom, Apparent oscillation peaks (average of spectrogram over time).

Before event detection and feature extraction, we removed signals that are suspected to be the results of ERPs. ERPs are prominent brief signals associated with external sensory stimuli. The wavelet transform might identify these transient signals as oscillations, obscuring our analysis. In order to remove these, we formed average ERP waveforms in supragranular, granular, and infragranular sink channels from an NHP A1 dataset, recorded during 50-dB auditory click stimulus presentations (Fig. 2). The wavelet transforms of these signals showed high spectral power. Both the supragranular and infragranular ERP responses have sharp peaks that last around 50 ms, producing a 20 Hz signal in the wavelet analysis. Similarly, the granular ERP response has a slower component that lasts for ∼100 ms, producing a 10-Hz response in the wavelet spectrogram.

ERP score was defined as the maximum normalized cross-correlation between that event and the average ERP from the same cortical layer (Fig. 2, top). An event with ERP score >0.8 with duration 75–300 ms (same range used for all layers; a loose constraint to reduce false negatives) was excluded from further analysis. ERP score was calculated for all NHP A1 events, but not for human STG where we lacked ERP data. We also excluded events with broadband frequency responses, likely from rapid-onset ERPs or recording noise. We defined logarithmic frequency span as Fspan = log(maxF/minF) using natural log, and excluded events with Fspan > 1.5 (2.17 octaves).

### Oscillation event (OEvent) detection and feature extraction

We extracted moderate/high-power spectral events using 7-cycle Morlet wavelets on nonoverlapping 10-s windows (Sherman et al., 2016; Neymotin et al., 2020). The 7-cycle Morlet wavelets were chosen to provide an adequate compromise between time and frequency resolution (Jones et al., 2009). We used linearly spaced frequencies (0.25-Hz frequency increments), ranging from 0.25 to 250 Hz, to compute the wavelets. The power time-series of each wavelet transform was normalized by median power across the full recording duration. We then applied a local maximum filter to detect peaks in the wavelet transform spectrogram. All local peaks were assessed to determine whether their power value exceeded a threshold. We used a moderate power threshold (4× median) to determine the occurrence of moderate-power to high-power events.

**Figure 3.**
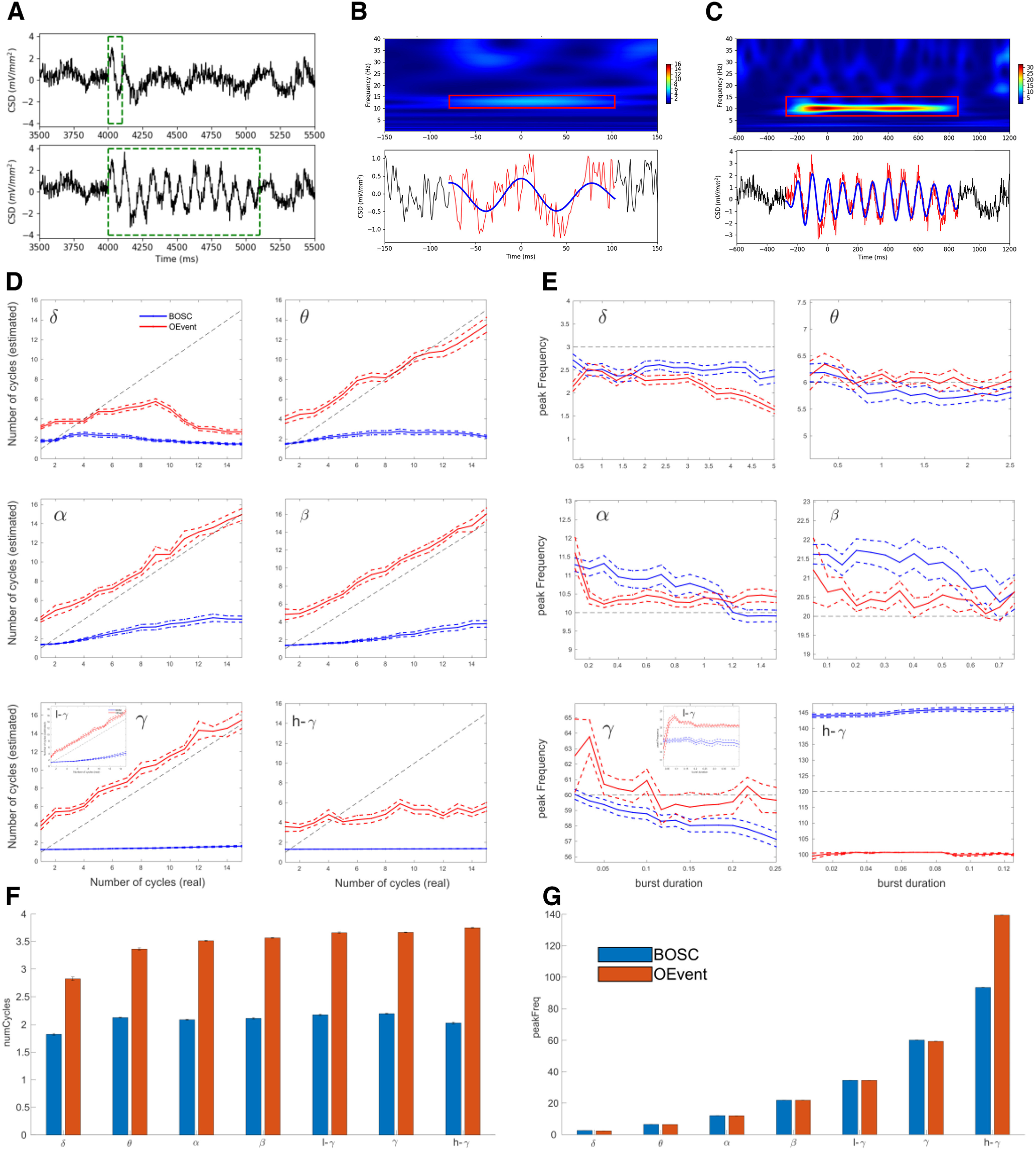
Validation of event detection algorithm. ***A***, Example of 1-cycle, 11-cycle simulated 10-Hz α signals (green bounding boxes) added to supragranular CSD; amplitude 1.5 mV/mm^2^. ***B***, 1-cycle signal from ***A*** was detected as two cycles. ***C***, 11-cycle detected as 11.6 cycles. ***B***, ***C***, Top, Wavelet transform spectrograms with detected event in boxes; bottom: raw (red), filtered (blue) signal. ***D***, Detected number of cycles as a function of the actual burst duration (dotted black line) for BOSC (blue) and OEvent (red). ***E***, Peak frequency detected was generally close to the frequency of the simulated signal (horizontal gray line) using both methods in most frequency bands but varied across different burst durations (SEM: dashed lines; insets in ***D***, ***E*** show the results for low-γ frequency band). ***F***, ***G***, Number of cycles and peak frequency detected in NHP A1 recordings using BOSC (blue) and OEvent (red).

**Figure 4.**
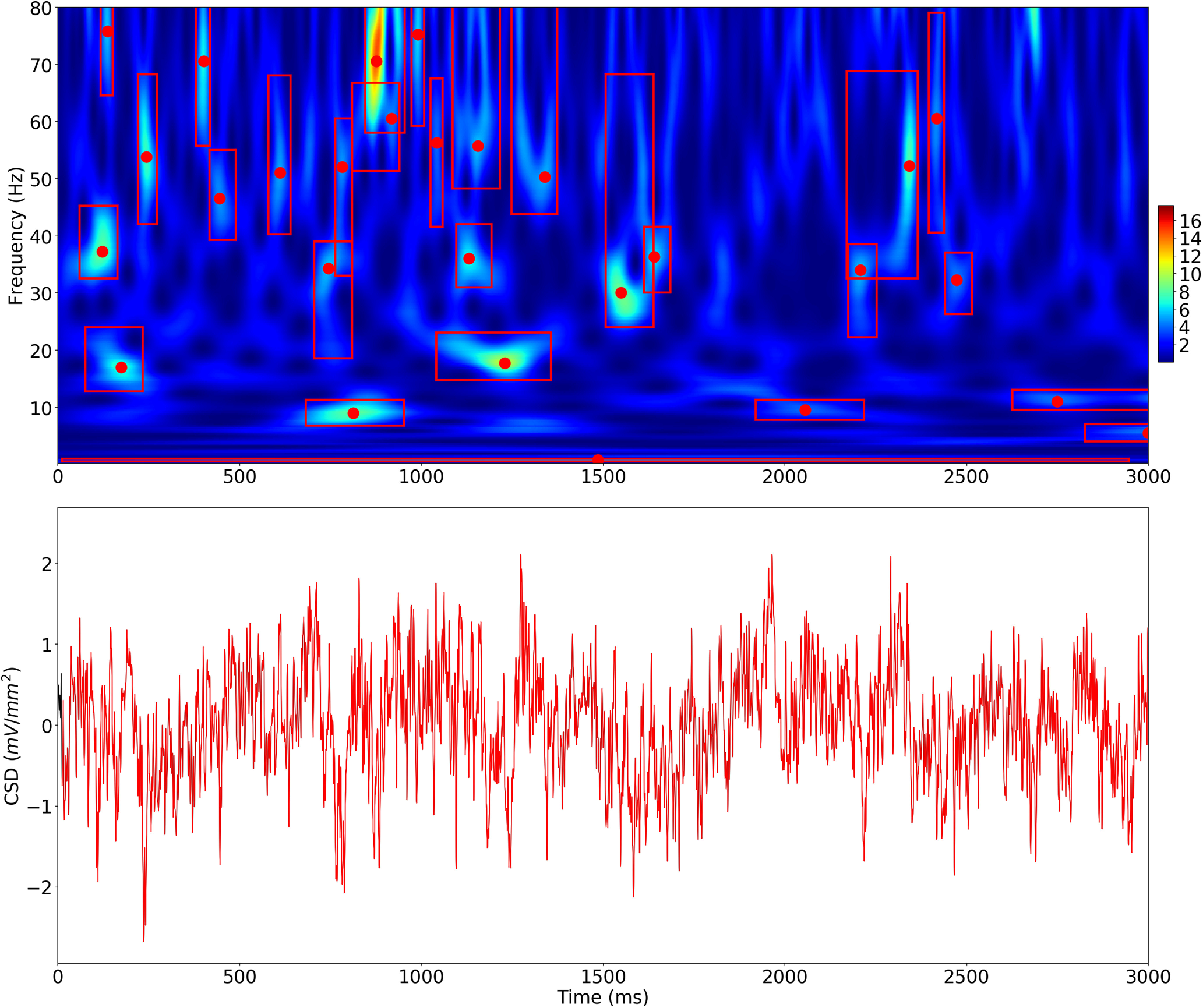
Oscillation events in at least one of the bands occupy the majority of recording time. Example from NHP supragranular A1. Oscillation events (red bounding boxes) occur in one or more frequency bands during this 3-s period. A long δ event is detected from *t* = 0 to near the end of this 3-s period (red box appears as line across bottom). Red dots show peak frequency; height of box indicates frequency spread. Normalized wavelet transform spectrogram (top) of signal shown at bottom (spectrogram values normalized by median power at a given frequency). This example shows events in δ, θ, α, β, low γ, and γ bands.

A local power peak in the spectrogram was defined within the local 3 × 3 window it was centered in, and exceeded the 4× median threshold of each individual frequency. The frequency and time bounds around that peak were determined by including time and frequency values before/after and above/below the peak frequency until the power value fell below the smaller of ½ maximum event amplitude and 4× median threshold. As shown in Figures 3, 4, this produces a bounding box around each oscillation event that can be used to determine frequency spread (minF to maxF), time span (start, stop), and peak frequency (defined as the frequency at which maximum wavelet power is detected). After the initial set of oscillation events is detected, we merge events when their bounding box overlapping area in the wavelet spectrogram exceeds 50% of the minimum area of each individual event. This allows continuity of events that are separated by minor fluctuations below threshold.

We then calculated additional features from this set of events. We calculated the number of cycles by multiplying the event duration by its peak frequency. We also filtered the underlying signals of each event using a zero-phase shift bandpass filter within the minF and maxF frequency ranges, defined on a per-event basis (as in bounding boxes shown in Figs. 3, 4).The zero-phase bandpass filter was calculated in Python using standard scipy signal library functions implemented in the open-source ObsPy signal processing library (https://github.com/obspy/obspy) that uses an infinite impulse response (IIR) butterworth filter, followed by forward and backward second-order cascaded IIR filters to preserve the phase. We calculated filter-match value *r*, defined as the Pearson correlation between this filtered signal and the raw signal, and used it as an index of how clearly the event oscillation is visible in the raw signal. Based on visual inspection of numerous waveforms, we suggest associating the following ranges of filter-match values with the corresponding qualitative assessments (0.0–0.25: weak; 0.25–0.5: moderate; >0.5: strong/high). Using the filtered signal also allowed us to count other features of the oscillation, including number of peaks (local maxima) and number of troughs (local minima). Number of peaks and the number of cycles were highly correlated. In figures showing waveforms of individual events, the 0 time alignment is taken as the wavelet transform 0 phase closest to the time of event threshold.

After extracting individual oscillation events, we classified them into the standard physiological oscillation frequency bands on the following intervals: δ (0.5–4 Hz), θ (4–9 Hz), α (9–15 Hz), β (15–29 Hz), low γ (30–40 Hz), γ (40–80 Hz), high γ (81–200 Hz). This classification was based on the frequency at which maximum power occurred during each event (intervals were open on the lower bound and closed on the upper bound).

We compared our OEvent method with the BOSC algorithm (Whitten et al., 2011) as shown in Figure 3.

### Phase-amplitude coupling (PAC) calculations

PAC was calculated by filtering the entire recording via convolution with a complex Morlet wavelet of width three and extracting the instantaneous phase and amplitude at the appropriate frequencies (f-phase = δ, 0.5–4 Hz; θ, 4–8 Hz; f-amplitude = γ, 30–200 Hz). Then, the instantaneous phase and amplitude were segmented based on the timing of the low-frequency events. Modulation index for segments containing low-frequency events and segments without any detected events was calculated (matched for number of segment and segment duration) using: $z\left(t\right)=Amp_{\gamma }\left(t\right)e_{\delta /\theta }{}^{i\varphi \left(t\right)}$. The modulation index is defined as the mean of z(t) and provides a metric of coupling between the two time-series. We then averaged the modulation index across segments with or without low-frequency events and compared the averages using a *t* test.

### IEI characterization

To measure rhythmicity across events from a given oscillation frequency band, we formed IEI distributions from the oscillation events which were not characterized as ERPs (see Results). We formed the intervals in two ways: (1) the interval between the time of peak power of the previous event to the time of peak power of the next event; (2) the interval between the end of the previous event to the start of the next event. After forming IEI distributions, we calculated its squared coefficient of variation (CV2). We also calculated the Fano Factor (FF) from the number of events in successive windows (defined below). The FF is defined as the variance to mean ratio of a random process in a certain time window, while the CV2 is defined as the variance over the squared mean. Both measures describe the extent of variability in relation to the mean. A Poisson distribution will have CV2 and FF values of 1, a more rhythmic process will have CV2 < 1, while a “bursty” process (multiple characteristic IEIs) will have CV2 > 1. CV2 values increased with the number of events in a time window, for both longer windows of analysis and higher frequency oscillations. To control for this, we varied window size for different frequencies (generally longer for slower frequencies) to produce a similar number of events per window (*N* ranging between 12–18, depending on frequency band and species). The empirically-determined window sizes used were 44.0, 30.0, 24.0, 10.7, 12.0, 3.6, 1.3 s for δ, θ, α, β, low γ, γ, and high γ frequency oscillations, respectively. Note that the window size for low γ events was longer than for β β events, since low γ events occurred less often. We used a one-sided Wilcoxon signed-rank test to determine that the measured average CV2 and FF values were lower than those of a Poisson process.

### Experimental design and statistical analyses

CV2 and FF were used as tests of rhythmicity. CV2 and FF are widely used measures to describe temporal variability of IEI distributions, with values intermediate between 0 (fully rhythmic and predictable) and 1 (fully noisy – Poisson distributed). To determine whether oscillation event IEI CV2 and FF values were lower than the values produced by a Poisson distribution, we used the one-sided Wilcoxon signed-rank test. Filter-match, also defined above, was the Pearson correlation between the filtered and raw signals from each oscillation event. We associated the following ranges of values with the corresponding qualitative assessments (0.0–0.25: weak; 0.25–0.5: moderate; >0.5: strong/high). We used a *t* test in the PAC analysis, as described above. For commonly used statistical tests we used the open-source numpy/scipy Python software libraries as well as the MATLAB software. The majority of the software was custom-written as described below. Full details of human and NHP experimental design are described in Datasets.

### Code/software accessibility

Python source code for our OEvent software package, for oscillation event detection and analysis, is available in an open-source repository at GitHub. The GitHub repository provides documentation on how to use OEvent to extract meaningful results. In the documentation on GitHub, we included an example CSD data signal recorded from NHP supragranular layers. The documentation shows how to extract the oscillation events, store them in the widely used Pandas dataframe format, and then select/view specific oscillation events using a custom-written event viewer. The event viewer was used to generate many of the figures in this manuscript.

## Results

The Results are organized as follows. First, we present a validation of our method using simulations, followed by specific examples of oscillation events in the time and frequency domains. Then, we summarize several characteristics of oscillation events for each frequency band and their intraevent variability. Finally, we look at shifts of events across different bands and cross-frequency interactions.

### Validation of OEvent package for oscillation event detection

We developed the Oscillation Event (OEvent) software package for the detection and analysis of electrophysiological oscillation event features. We validated OEvent by measuring its accuracy in detecting the number of cycles and the peak frequency in a simulated dataset of sinusoidal signals in several frequency bands and compared the results with a different oscillation detection method which is based on a power and duration threshold termed Better Oscillation Detection (BOSC; Whitten et al., 2011; Hughes et al., 2012). Oscillation length was varied from 1 to 15 cycles with a sufficient interval between event initiation to allow for event detection. For each band, oscillation event signals were superimposed on an NHP auditory cortex supragranular CSD signal to provide realistic physiological background signal statistics (Fig. 3).

As expected, a single cycle α event was difficult to detect correctly (Fig. 3*A*,*B*). In the example shown in Figure 3*A*, top, a single cycle α event was added to the CSD signal. The random placement of the single cycle event was such that it was followed by an additional cycle of the same frequency that appeared in the CSD data, something that can readily occur randomly in this oscillation rich environment. Unsurprisingly, OEvent detected 2.3 cycles and overestimated the peak frequency relative to the added cycle (12.75 Hz). An additional factor for overestimation of both frequency and number of cycles were the sharp discontinuity transients at the beginning and end associated with superimposing signal on background.

OEvent was more accurate in estimating the properties of prolonged oscillation events: an 11-cycle α event was calculated as 11.6 cycles, with frequency calculated as 10.25 Hz (Fig. 3*A*,*C*). Estimation accuracy for all frequency bands are shown in Figure 3*D* (number of cycles) and *E* (peak frequency) for both BOSC (blue) and OEvent (red). In the δ band, both methods struggled to accurately estimate the number of cycles and the peak frequency, specifically for longer durations. At higher frequencies OEvent performed better at estimating the correct number of cycles while both methods performed approximately the same in estimating the peak frequency of the oscillations.

Accuracy across all cycle lengths had an RMS error between 1.45 and 2.46 for θ-γ bands across all cycle durations. For the δ and high γ ranges, RMS had higher values of 5.97 and 4.7, respectively, specifically for higher number of cycles (more than six cycles; Fig. 3*D*). As in the case of Figure 3*A*, in most frequency bands, the number of cycles was typically overestimated for lower-cycle events and performance improved as the number of cycles in the simulated signal increased (except for δ and high γ bands). Frequency estimation accuracy also varied with the number of cycles (Fig. 3*E*). At a low number of simulated cycles, the peak frequency was more strongly overestimated, which also contributed to overestimation of number of cycles, based on duration and frequency. As in the case of the number of cycles, estimation of the peak frequency had the largest errors in the δ and high γ ranges. In addition, we compared the results of the two methods using NHP A1 recordings from two animals (see Materials and Methods). As in the case of the simulated data, the number of cycles detected by OEvent seemed to be higher in all frequency bands and the peak frequency was approximately similar for both methods with a higher frequency detected by OEvent in the high γ range (Fig. 3*F*,*G*).

### Characterization of oscillations

8.13 h of linear array (23 channels spanning 2.3 millimeters encompassing all cortical layers) NHP A1 recordings from four animals were converted to CSD time-series to estimate neuronal ensemble transmembrane currents (Lakatos et al., 2016). In this dataset, we detected over 1.9 million putative oscillation events using the OEvent method across all recording channels, after eliminating ERP-like events (see Materials and Methods). We also analyzed 37 min of human STG iEEG recordings (five subjects; 43 923 oscillation events). We performed the analyses on the iEEG without calculating a CSD. Re-referencing iEEG signals using a bipolar referencing scheme produced similar results. We focused on characterization of oscillation events, as periods in the wavelet spectrogram with moderate to high power at particular frequencies (defined as above a threshold of 4× median power; see Materials and Methods). Events were classified according to traditional frequency bands, with some auditory system specific adjustments: δ (0.5–4 Hz), θ (4–9 Hz), α (9–15 Hz), β (15–29 Hz), low γ (30–40 Hz), γ (40–80 Hz), high γ (81–200 Hz). Event frequency was defined based on the frequency at the point of maximum power during the event. In the following section, we provide raw examples of oscillation events in both humans and NHPs and qualitatively discuss their properties to allow the reader and the user of OEvent to get familiar with the representation of the oscillation events in the time and frequency domains. Quantitative assessment of specific characteristics of oscillation events is presented in the next section.

Using OEvent, we found that the majority of the recording time contains detectable oscillations in at least one of the studied frequency bands, indicating that oscillations might be a key component in A1 dynamics (Fig. 4). While the majority of the activity in individual bands is not detected as having oscillation events, oscillation events in one or more frequency bands were detectable during ∼90% of the total recording duration (88.1%, 89.4%, 89.0% for the three NHP A1 locations; 89.6% in human STG iEEG). Results were consistent across layers and between NHP and human recordings. Oscillations were typically overlapping or nested.

**Figure 5.**
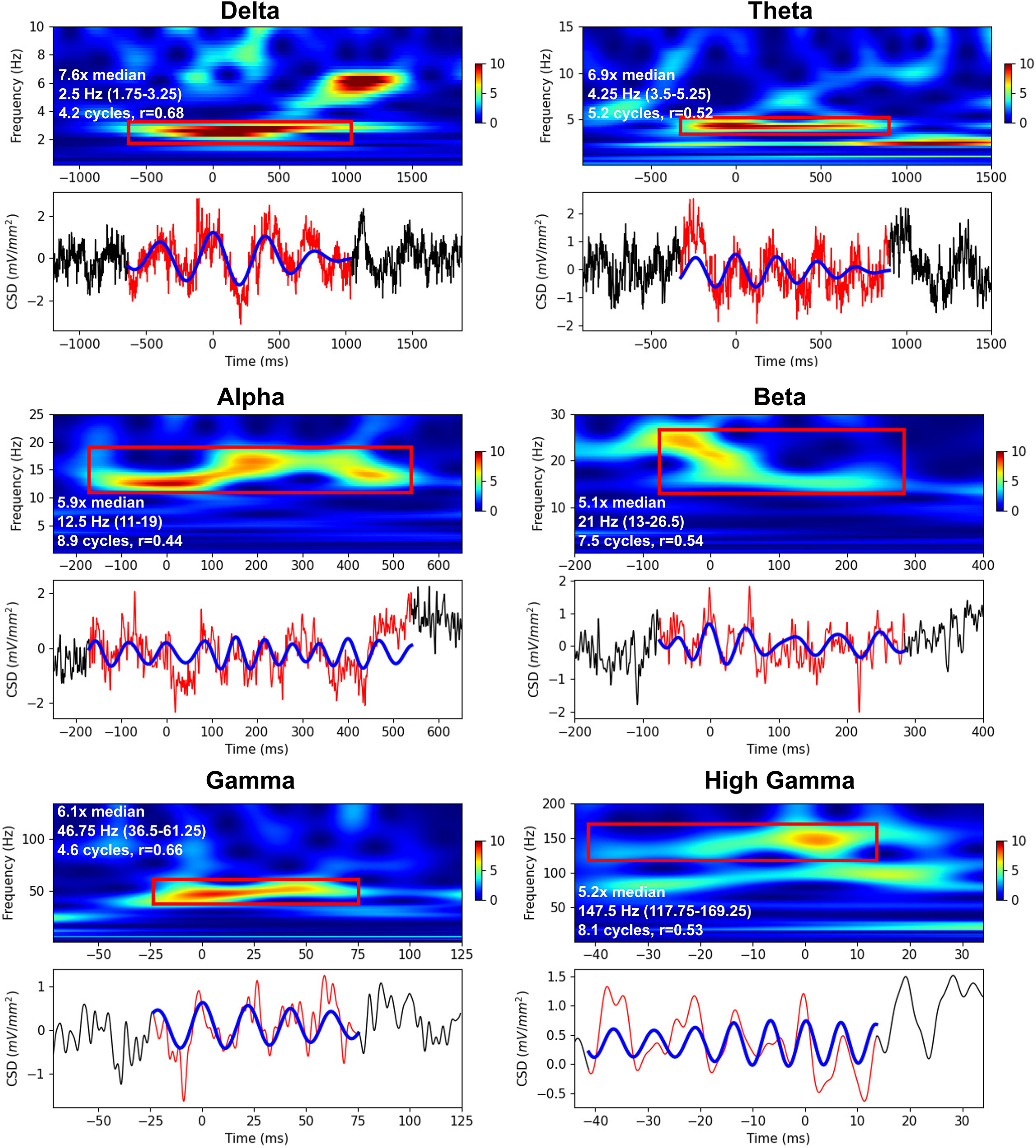
Examples of oscillation events from NHP A1 supragranular layer. Normalized Morlet wavelet spectrograms demonstrating individual events (red bounding-box) with raw (red) and filtered (blue) waveforms below (black trace: period outside of detected oscillation). Note that x- and y-scales differ for different bands; spectrogram power (color) is in median normalized units. White text in the spectrograms specifies the events’ power relative to the median, the peak frequency of the event (and frequency range), the number of cycles, and the correlation value between the raw and filtered waveforms (filter-match).

Figure 5 shows a few examples of oscillation events detected in NHPs data. Oscillation events varied widely in appearance, frequently with crescendo-decrescendo patterns. Many events also showed a pattern of frequency change – up, down, or U-shaped. For example, the α event in Figure 5 maintained relatively constant power but first increased and then decreased in frequency, as can be seen in the time domain as well as in the spectrum. Despite the relatively broad spread of frequency, this α event is clearly continuous, qualifying it as a single oscillation event. The β event in the same figure showed a different pattern: an abrupt frequency reduction concomitant with power reduction. There was also variation of frequency across events within a frequency band. These observations demonstrate an important aspect of neuronal oscillations that emerges from studying these fluctuations using single-trial (or nontask related) ongoing recordings: oscillation events, even within the same band, might show different characteristics that may shed light on their functional role and the mechanisms generating them.

As in prior studies (Lakatos et al., 2008, 2016), δ rhythm dominated in power as well as in “active time” (proportion of the recording time that δ rhythms were present). In both the δ and θ cases, a typical nesting of fast oscillations within the slower oscillation was seen, explaining the relatively low filter-match (correlation between filtered and raw signal; for quantification, see Materials and Methods).

**Figure 6.**
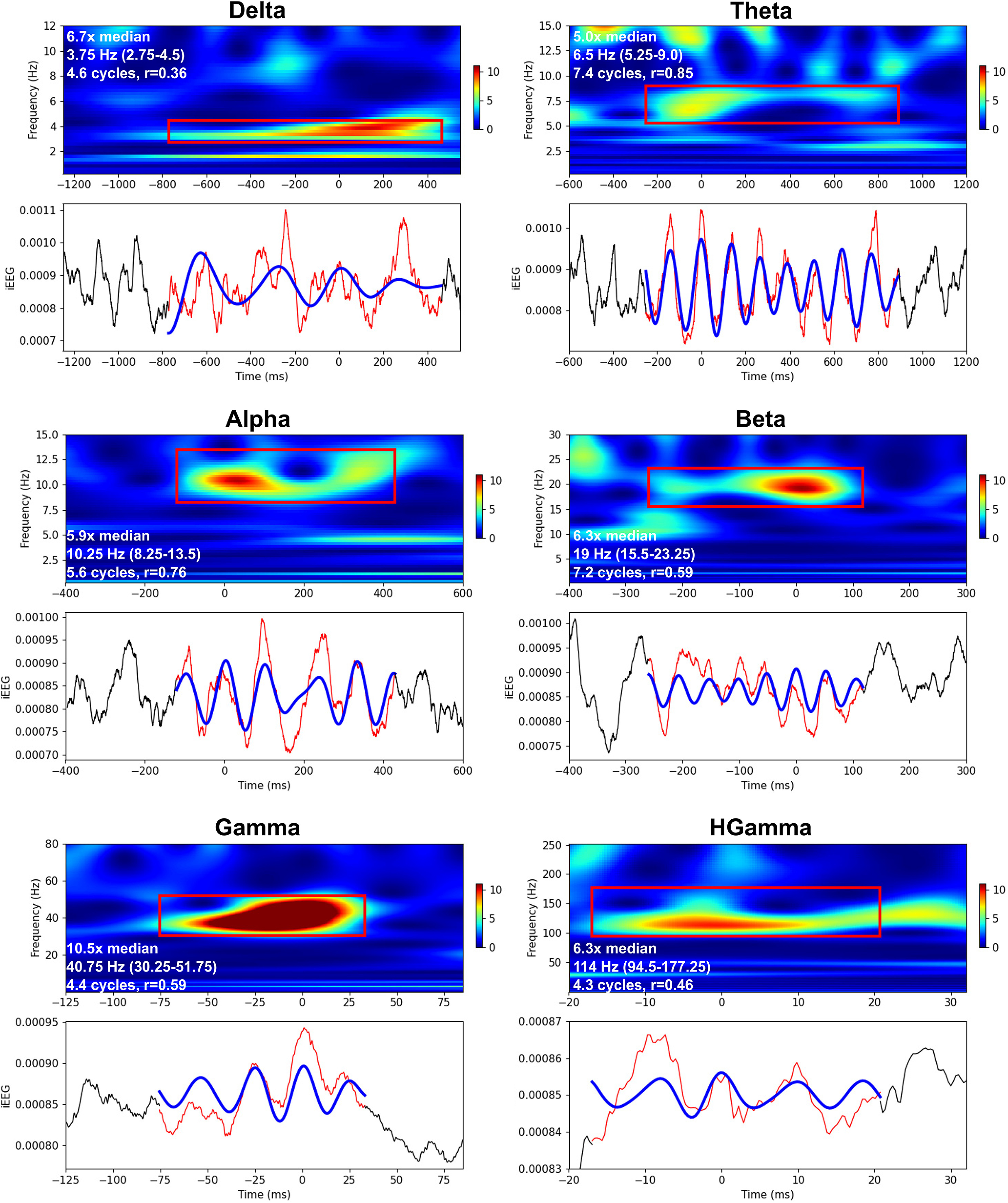
Examples of oscillation events from human STG iEEG. Normalized Morlet wavelet spectrograms demonstrating individual events (red bounding-box) with raw (red) and filtered (blue) waveforms below [x-y-scales differ for different bands; spectrogram power (color) is in median normalized units; iEEG time-series values are in units of Volts]. Time of 0 ms corresponds to the wavelet phase of 0 radians (local maxima) closest to the event’s peak power at threshold detection. White text in the spectrograms specifies the events’ power relative to the median, the peak frequency of the event (and frequency range), the number of cycles, and the correlation value between the raw and filtered waveforms (filter-match).

The characteristics of human oscillation events were similar to those of NHP (Fig. 6). Oscillation events in the human iEEG were clearly detected across all physiological oscillation bands, with multiple cycles and strong correspondence between raw and filtered waveforms. As with the NHP results, events showed intraevent shifts in frequency and amplitude, for example the α event in Figure 6 shows a central frequency dip.

**Figure 7.**
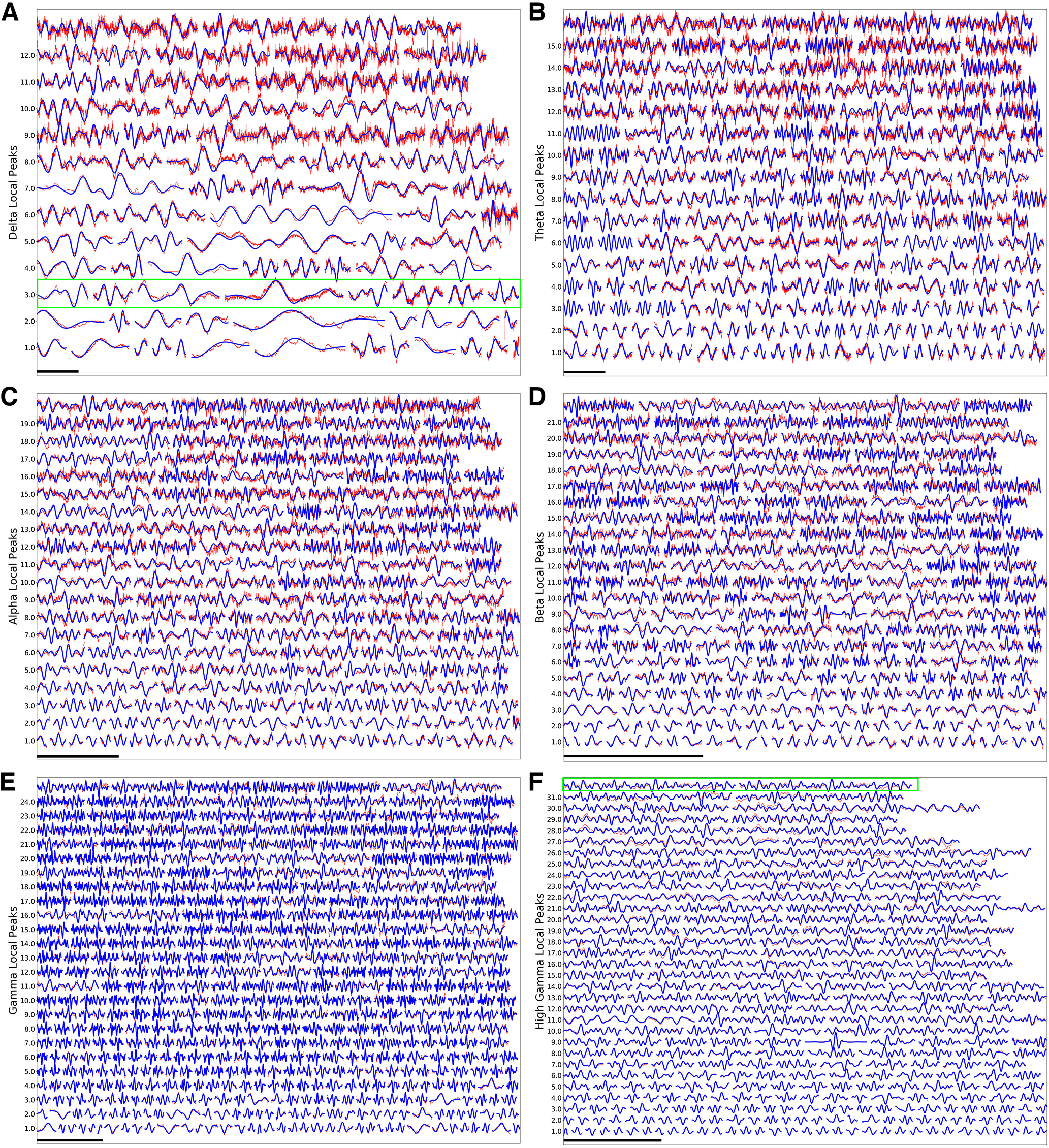
Individual oscillation events from NHP A1. δ (***A***), θ (***B***), α (***C***), β (***D***), γ (***E***), high γ (***F***). In each case, vertical axis arranges waveforms with decreasing numbers of cycles from top to bottom; each row organizes waveforms with decreasing filter-match between raw and filtered signal from left to right. Horizontal scale bars 1 s except: ***E***, 200 ms; ***F***, 100 ms. Examples in green bounding boxes in ***A***, ***F*** are described in the text. Note that waveforms are normalized to allow easier visual comparison.

To better illustrate the time-domain variability in waveform and duration of oscillation events across different bands, Figure 7 shows examples of raw and filtered data from NHP A1 neuronal activity sorted by frequency band, with events organized from top-to-bottom by decreasing number of cycles, and left-to-right by decreasing filter-match (Pearson correlation between an event’s filtered and raw signals; see Materials and Methods). Oscillations are easier to visually detect in the case of high filter-match. Direct inspection of many such examples allowed us to verify that the algorithm detected “reasonable-looking” oscillations. The oscillations shown here had as many as 32 local peaks (Fig. 7*F*, two examples in green bounding box). Combinations of oscillations which co-occurred within a specific event produced differences in waveforms (Fig. 7*A*, green bounding box). Importantly, the variability in waveform shapes and oscillation event properties suggests different circuit mechanisms for the production of individual events and might also account for different internal cognitive processes associated with oscillation events within a specific band.

### Oscillation event features and variability

**Figure 8.**
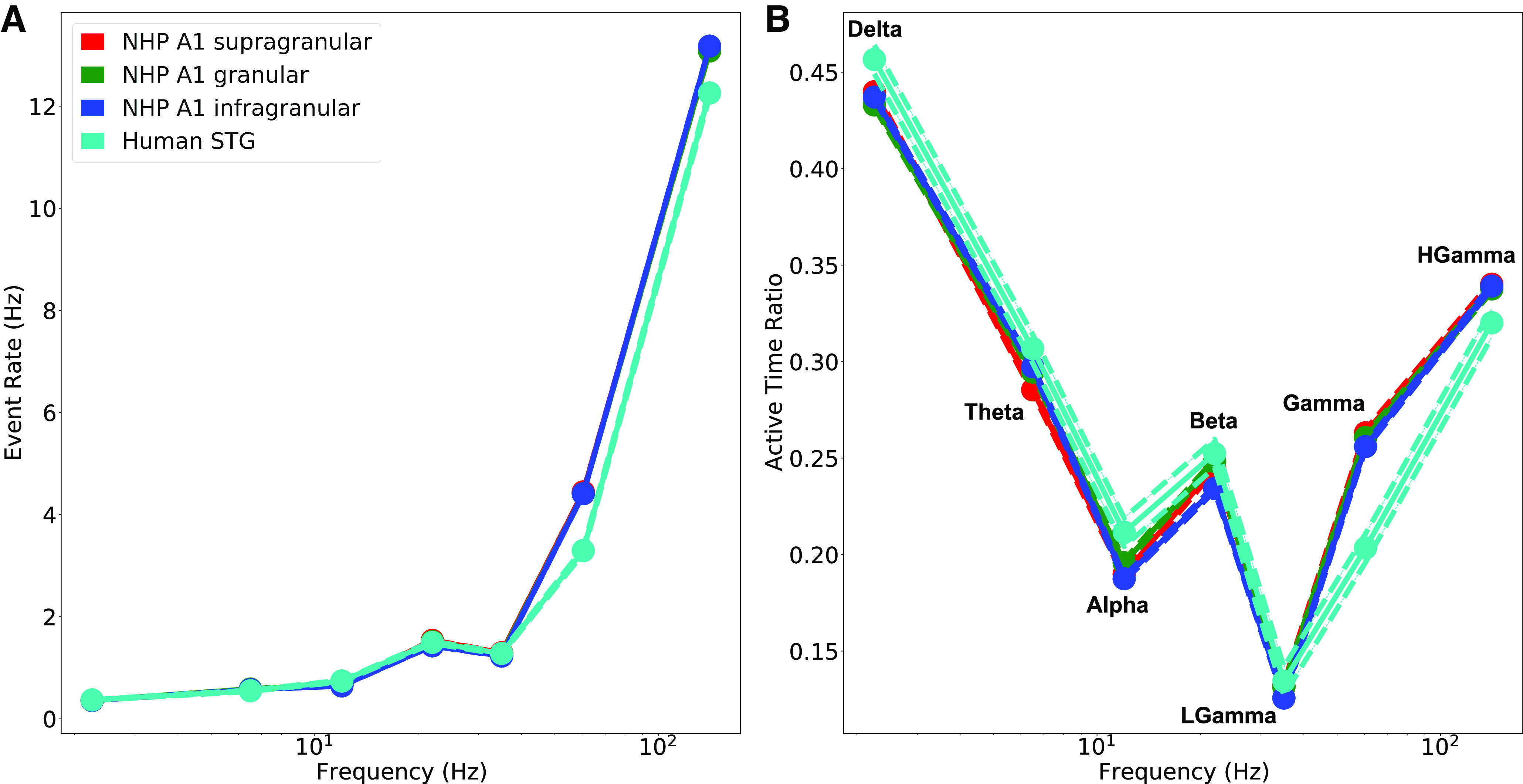
Oscillation event rates and active time varied by frequency band. ***A***, Higher frequency events are more frequent. ***B***, ATR: lower and higher frequency events fill much of the recording duration (HGamma: high-γ bands; mean ± SEM in both ***A***, ***B***; see Extended Data Table 8-1, Table 8-2).

Next, we quantified the rate of oscillation events at each frequency band and their overall proportion in the recording duration (active time ratio; ATR) for NHPs and human patients. Lower frequency events will tend to occur more rarely because of their longer duration. Therefore, it was not surprising that higher frequency events occurred most often (Fig. 8*A*). However, longer event durations for δ oscillations produced the longest overall ATR for the δ band (Fig. 8*B*). ATR values ranged from 0.13 to 0.46, with the same pattern seen in all NHP cortical layers, and in human STG: highest for δ, decreasing for θ, α, and low-γ, then increasing for β, γ, and high γ. The ATRs add up to >1, >100%, because of the oscillation overlap seen in Figure 4. Overlap was often because of nesting of a high frequency oscillation in a lower frequency event. For example, γ and high γ (Hgamma) both had high ATRs because of nesting in δ or θ bands (also see co-occurrence analysis in Fig. 12*A*). This pattern was observed both when using longer window sizes for slower oscillation frequencies compared with faster oscillation frequencies, and when using the full recording duration for the event rate calculations.

10.1523/ENEURO.0281-21.2022.t8-1 Extended Data Table 8-1 Average event rate (Hz) ± SEM, and event count in parentheses, for the different physiological oscillation frequency bands. A1 Supra, A1 Gran, and A1 Infra are from NHP A1 supragranular, granular, and infragranular sink channels, respectively. STG is from human iEEG recorded in supratemporal gyrus. Download Table 8-1, DOCX file.

10.1523/ENEURO.0281-21.2022.t8-2 Extended Data Table 8-2 ATR for the different physiological oscillation frequency bands. Mean value is presented (SE was negligible). A1 Supra, A1 Gran, and A1 Infra are from NHP A1 supragranular, granular, and infragranular sink channels, respectively. STG is from human iEEG recorded in supratemporal gyrus. Download Table 8-2, DOCX file.

**Figure 9.**
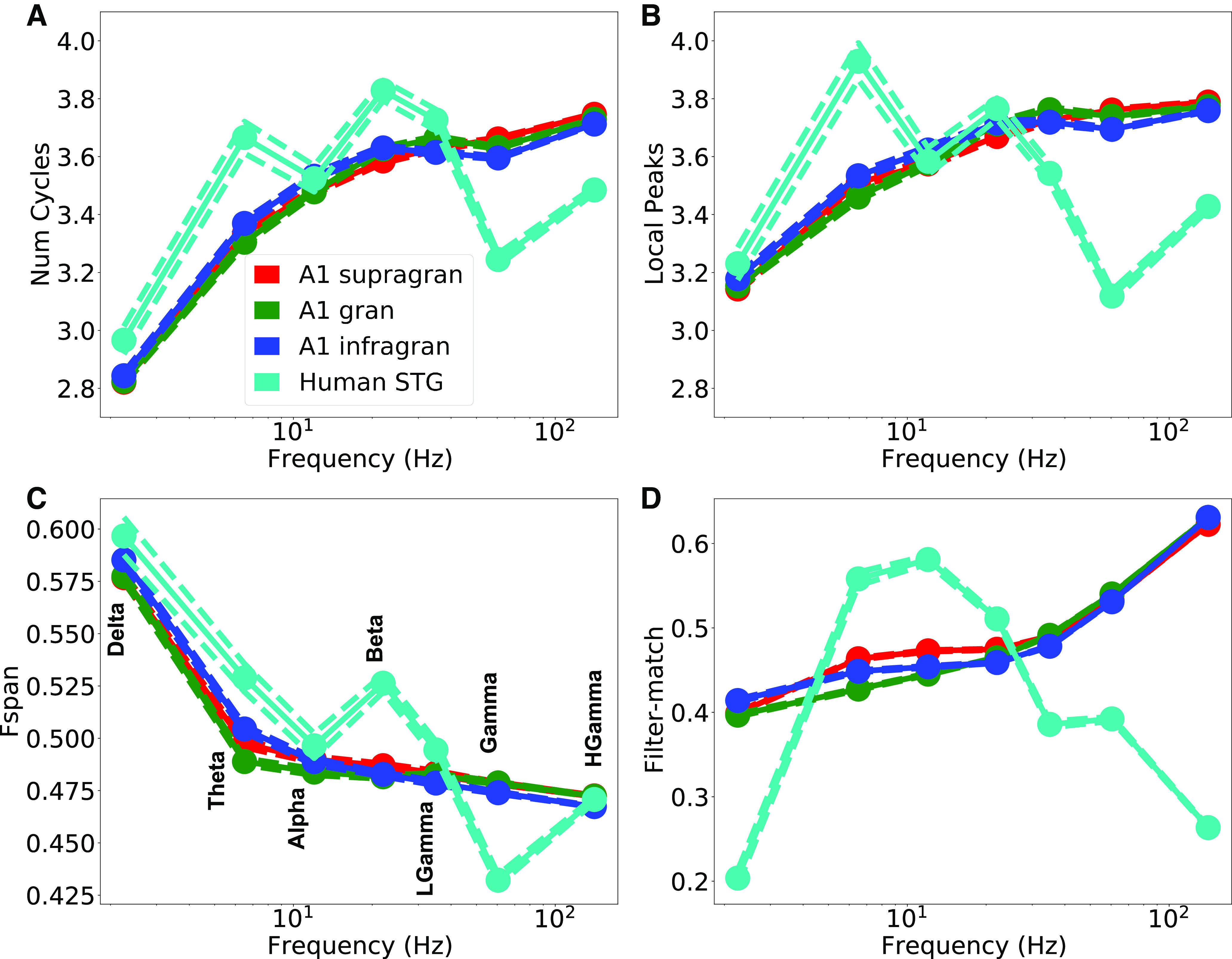
Event features. ***A***, Number of cycles. ***B***, Number of local peaks in the time domain of the filtered waveform. ***C***, Intraevent Fspan = log(maxF/minF); 0.7 is freq doubling. ***D***, Filter-match *r* value (see Extended Data Tables 9-1, 9-2, 9-3, 9-4 for comparisons).

While most oscillation characteristics were consistent across NHP layer locations, they were somewhat different when compared with human recordings (Fig. 9; see Extended Data Table 9-1, Table 9-2). The number of detected cycles averaged 3–4 (range 1–44), increasing from δ to high γ (Fig. 9*A*). Time-domain count of local peaks closely matched the number of calculated cycles, demonstrating the accuracy of the measures taken in the wavelet domain (Fig. 9*B*). All oscillation frequency bands above δ had numerous events with >10 cycles in both NHP and human recordings. We defined intraevent frequency span as Fspan = log(maxF/minF) with minF,maxF the event’s minimum and maximum frequencies, respectively. Bandwidth was consistent across bands with intraevent Fspan showing maxF ∼65% higher than minF (Fig. 9*C*; antilog(0.5) = 1.65). A higher number of intraevent frequency shifts were seen in the δ band. Interestingly, human β was broader in bandwidth, and human γ tighter, compared with the values in NHP. Quality of filter-match increased with frequency in NHP (Fig. 9*D*). We believe that filter-match was worse at the lower frequencies because of overlapping higher frequency nested oscillations, that “distorted” quasi-sinusoidal wave shapes. This filter-match tendency differed in the human recordings, where high frequencies were poorly fitted by the filtered waveforms. The difference may be a technical consequence of more volume conducted activity, and thereby summation in iEEG recordings, but could also be a consequence of more intricate circuitry producing high frequency activity in humans versus NHP. Future invasive recording/modeling studies will be needed to decipher this issue.

10.1523/ENEURO.0281-21.2022.t9-1 Extended Data Table 9-1 Cycles Per Event for the different physiological oscillation frequency bands. Range and mean ± SEM, separated by semicolon (;). A1 Supra, A1 Gran, and A1 Infra are from NHP A1 supragranular, granular, and infragranular sink channels, respectively. STG is human iEEG signals recorded from supratemporal gyrus. Download Table 9-1, DOCX file.

10.1523/ENEURO.0281-21.2022.t9-2 Extended Data Table 9-2 Number of local peaks in filtered waveforms for the different physiological oscillation frequency bands. Range and mean ± SEM are presented, separated by semicolon (;). A1 Supra, A1 Gran, A1 Infra are from NHP A1 supragranular, granular, and infragranular sink channels, respectively. STG is human iEEG signals recorded from supratemporal gyrus. Download Table 9-2, DOCX file.

10.1523/ENEURO.0281-21.2022.t9-3 Extended Data Table 9-3 Logarithmic frequency bandwidth (Fspan) for the different physiological oscillation frequency bands. Values are mean ± SEM. A1 Supra, A1 Gran, and A1 Infra are from NHP A1 supragranular, granular, and infragranular sink channels, respectively. STG is human iEEG signals recorded from supratemporal gyrus. Download Table 9-3, DOCX file.

10.1523/ENEURO.0281-21.2022.t9-4 Extended Data Table 9-4 Correlation between filtered and raw signals (filter-match) for the different physiological oscillation frequency bands. Values are mean ± SEM. A1 Supra, A1 Gran, and A1 Infra are from NHP A1 supragranular, granular, and infragranular sink channels, respectively. STG is human iEEG signals recorded from supratemporal gyrus. Download Table 9-4, DOCX file.

A similar comparison was performed for multinuint activity (MUA) signals which tend to show higher number of cycles and lower filter-match values, but because of technical reasons (i.e., MUA is a noisier signal than LFP or iEEG because of environmental noise, movement, etc.), it is difficult to reliably interpret the observed differences between MUA and CSD characteristics from a physiological point of view. Nevertheless, the results of this analysis are shown in Extended Data Figure 9-1, demonstrating that the OEvent method can also be applied to the MUA signal.

10.1523/ENEURO.0281-21.2022.f9-1 Extended Data Figure 9-1 Comparison of features for oscillation events detected from NHP CSD (blue) and NHP multiunit activity (MUA; red). ***A***, While there is a higher number of cycles detected in MUA, the same pattern of increasing number of cycles with higher oscillation frequency is evident. ***B***, Filter-match was higher across the oscillation frequencies for CSD. Download Figure 9-1, EPS file.

**Figure 10.**
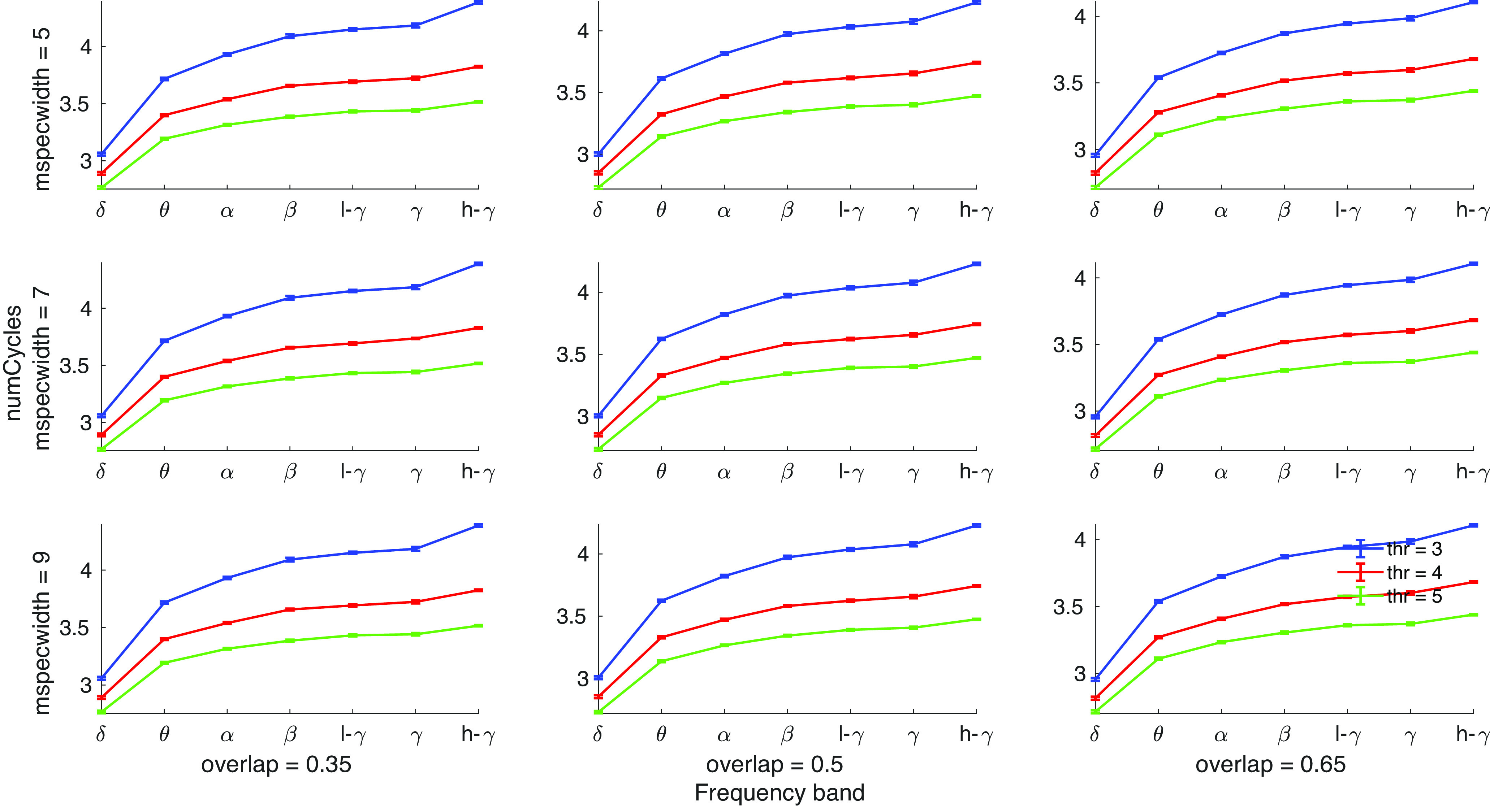
Parameters of OEvent. Influence of different parameter combinations on the detected number of cycles for each frequency band. The results are averaged across all recordings and cortical layers. Error bars indicate SEM. Each row corresponds to a different wavelet width (mspecwidth = 5, 7, 9), and each column corresponds to a different threshold for merging overlapping events (overlap = 0.35, 0.5, 0.65). Colored lines indicate different detection thresholds. Higher detection thresholds resulted in lower detected number of cycles for all bands (*t* test, *p* < 0.01 for all bands, FDR corrected). No significant differences were observed between different wavelet widths or overlap thresholds within any of the bands.

There are several key parameters that might be adjusted when using OEvent. To test the influence of specific parameters, we systematically studied different parameter combinations and their effects on the characteristics of the oscillatory events. Specifically, we modified the detection threshold, the width of the wavelets used, and the overlap threshold to merge overlapping events. Figure 10 shows the effects of modifying these parameters on the detected number of cycles for each frequency band. Since no significant differences were found between cortical layers, we averaged the results across layers. As expected, we found that the variable that has the strongest influence on the characteristics of the oscillation events is the detection threshold (i.e., higher detection threshold results in a lower number of cycles for all wavelet widths and overlap thresholds). In order to detect oscillation events, one must determine a threshold for detection and the thresholds we selected have been previously used by other researchers to detect similar signals (Sherman et al., 2016). The two other parameters we compared did not show significant effects on the studied characteristics.

**Figure 11.**
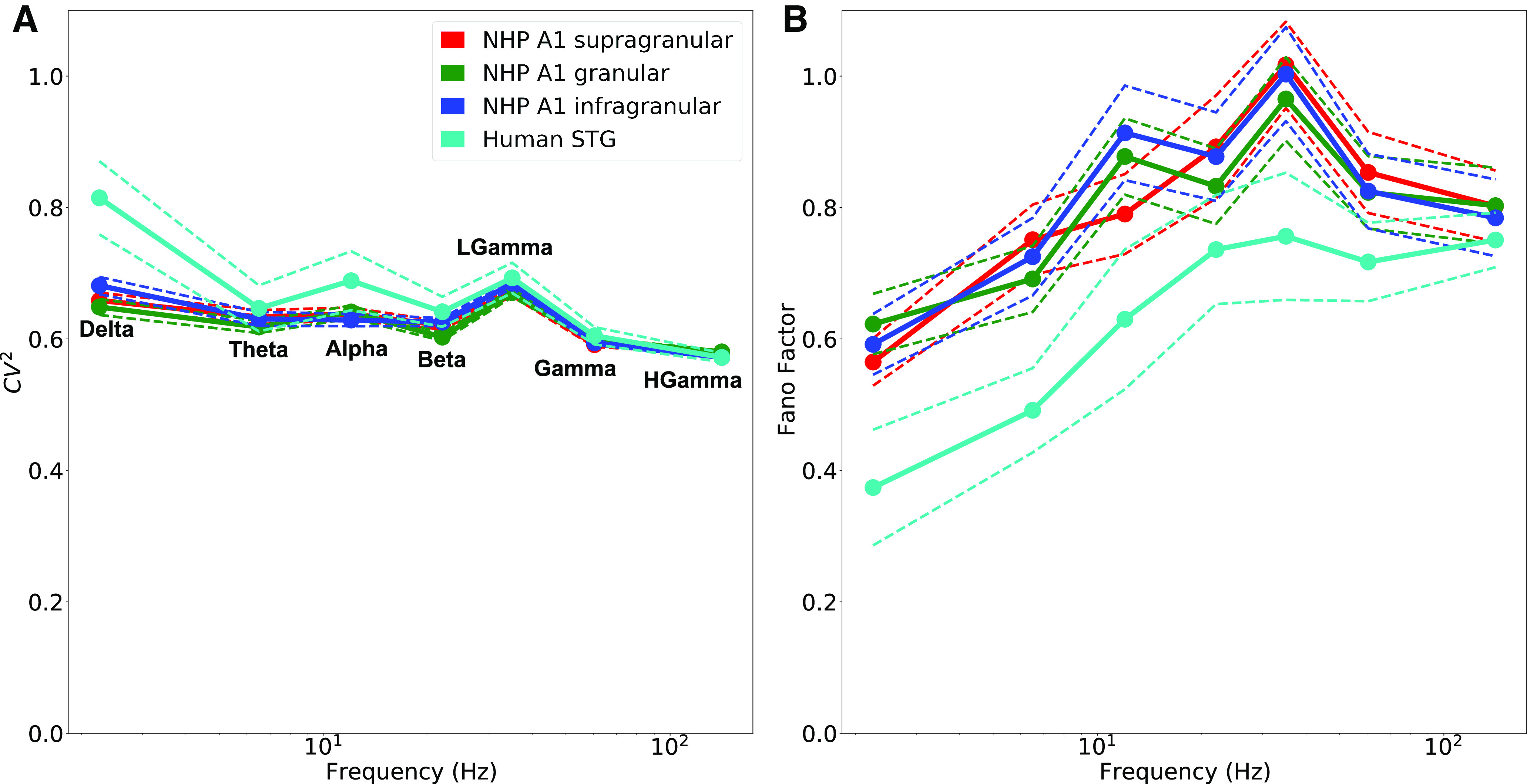
IEIs suggest rhythmic recurrence in all bands. ***A***, CV2 values (mean and SEM) in all bands < 1 (*p* < 0.05, one-sided Wilcoxon signed-rank test). ***B***, FF (mean and SEM) in all bands except for LGamma < 1 (*p* < 0.05, one-sided Wilcoxon signed-rank test).

Events occurred with some degree of rhythmicity, suggesting a rhythmic occurrence of these oscillation-events within windows of time (Fig. 11). The testing windows of 44.0, 30.0, 24.0, 10.7, 12.0, 3.6, and 1.3 s for δ, θ, α, β, low γ, γ, and high γ were sufficient to allow ∼16 events per window (see Materials and Methods). IEIs within a particular band were measured as times between event peaks, or by event initiation or termination (similar results). CV2 and FF were used as tests of rhythmicity. CV2 and FF are widely used measures in the literature of spike trains to describe their variability, with values intermediate between 0 (fully rhythmic and predictable) and 1 (fully noisy – Poisson distributed). Additional details about these measures are provided in Materials and Methods.

Average CV2 values were all substantially lower than 1.0 (*p* < 0.05, one-sided Wilcoxon signed-rank test; Fig. 11*A*), demonstrating that events occurred across time with some rhythmicity. FF values were also consistent with this hypothesis, with mean values lower than 1.0 (*p* < 0.05, one-sided Wilcoxon signed-rank test; Fig. 11*B*), with the exception of the LGamma band in NHPs, which had FF values slightly higher than 1.

### Oscillation events across bands

**Figure 12.**
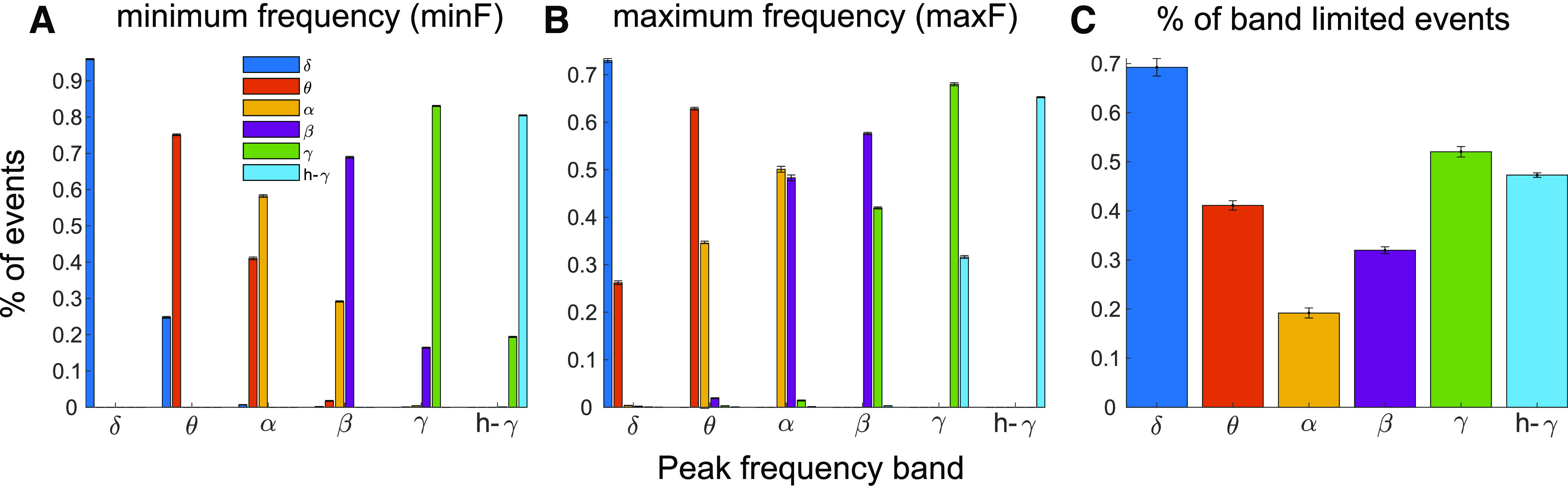
Individual oscillation events are categorized by the frequency of maximum amplitude (peak frequency; *x*-axis) but may spread to lower or higher frequency bands. ***A***, ***B***, % of oscillation events with minimum and maximum frequency in a specific band. ***C***, % of oscillation events that are band-limited, defined as having both minF and maxF in the same band as the event’s peak. In some frequency bands (i.e., δ, γ, and high γ) individual events tend to be more band limited (***C***), while in other bands (i.e., θ, α, and β) as many as 60−80% of the events spread to adjacent bands (*x*-axis location indicates categorization of an oscillation event based on its peak frequency; color indicates which frequency band an oscillation event spreads to, e.g., in ***A***, ∼75%, 25% of θ events have a minimum frequency in the θ, δ bands, respectively).

A common practice in the study of neuronal oscillations, which we adopted as well, is to divide oscillatory activity into specific bands of interest (i.e., δ, θ, α, β, low γ, γ, high γ). However, an oscillation event might span several of these bands. Thus, for each band of interest, we tested the percentage of band-limited events and the percentage of events that occupy multiple bands. To this end, we first defined the events based on the frequency at the peak amplitude of the event. We then tested whether the detected minimal and maximal frequency of each event (minF and maxF, respectively) were both in the same band as the peak (band-limited), or whether either crossed into other frequency bands. The results of this analysis are shown in Figure 12 for each band. Since all cortical layers showed similar results, we combined the data across layers in NHPs. 15–25% of the events with a peak in the θ, β, γ, and high γ bands spread into lower frequencies, whereas ∼40% of the events with a peak in the α range spread into the θ range as well. In addition, ∼25–35% of events with a peak in the δ, θ, and γ bands spread into higher frequencies, whereas 40–50% of α and β events spread into the β and γ band, respectively. In total, ∼40–70% of δ, θ, γ, and high-γ events were band-limited, while only 20–30% of events in α and β bands were band-limited. While some of this variability across bands can be explained by the variability in bandwidth, this result indicates that some frequency bands tend to be more band-limited than others in A1 ongoing recordings. Note that in this analysis we used a combined γ band (30–80 Hz) since low-γ (30–40 Hz) is fairly narrow, which led to low-γ having very few band-limited events with too many transitions from β to low-γ and from low-γ to γ (data not shown).

**Figure 13.**
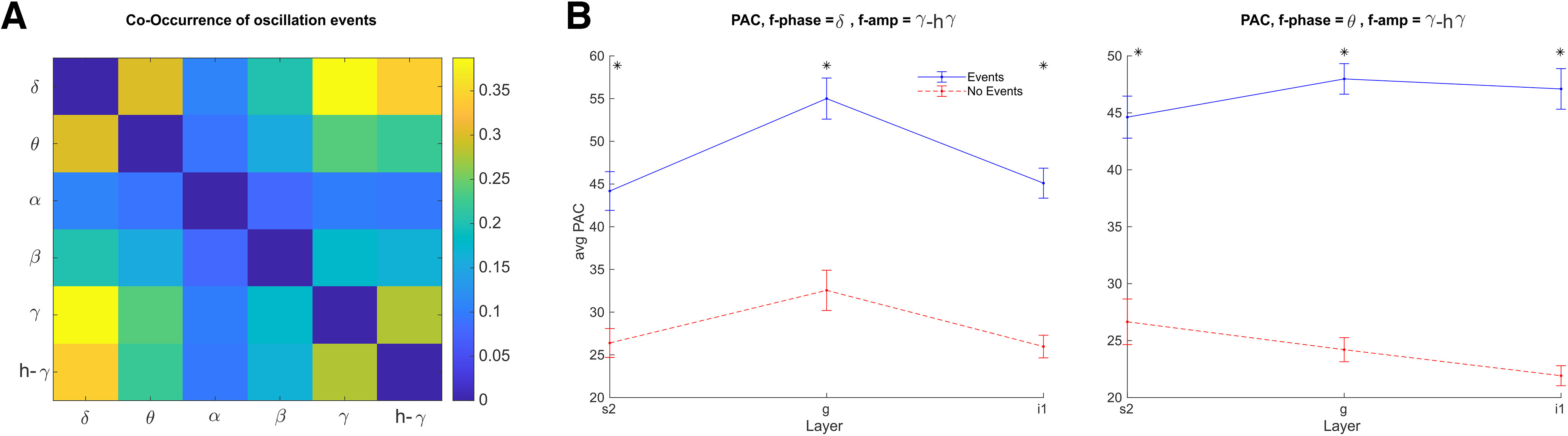
Co-occurrence of oscillation events and PAC. ***A***, Oscillation events in the δ-θ range had higher probability of occurring together with γ-high γ events compared with other frequency band combinations. ***B***, PAC between δ (left) or θ (right) events and γ/h-γ events during periods of low-frequency events (blue) and periods without any detected events (red). PAC in all layers increases during periods of low-frequency events. (Note that just as in the analysis shown in Figure 9, here we used a γ band spanning 30–80 Hz, to allow accurate measurement of cross-frequency interactions in a wide enough band.)

Neuronal oscillations have been suggested as foundational in auditory processing by “chunking” the auditory stream into temporal units for further processing (Ghitza, 2017; Rimmele et al., 2021). Specifically, oscillations in the δ-θ and γ range seem to be engaged in tracking the dynamics of auditory information (Giraud and Poeppel, 2012; Teng et al., 2017). To further test the hypothesis that resting state auditory cortex dynamics are dominated by oscillations within these bands, we tested whether events in different frequency bands have higher probability of occurring together in the NHP auditory cortex. For each pair of frequency bands, we calculated the probability of co-occurrence as the number of events that occurred together (i.e., at the same time) out of the total number of events in these bands. Since the results were similar across the different cortical layers, we pooled the data from the different layers together. As predicted, the highest probability of event co-occurrence was found between δ-θ and γ bands (Fig. 13*A*). Events in the α range had the lowest probability of co-occurrence with any other frequency band. These results indicate that transient oscillation events within the δ-θ and γ frequency bands are interconnected in the auditory cortex even in the absence of any auditory stimuli. This might indicate distinct δ-θ versus α-β dominated brain states, as one of our former (Lakatos et al., 2016) and another study demonstrated (Teng et al., 2017).

To further test the interaction between δ-θ and γ oscillations, we calculated the PAC between these bands during δ and θ oscillation events and compared them to segments without oscillation events from the same recordings. Both δ and θ bands showed an increase in PAC with γ band oscillations during the oscillation events compared with no-event segments (*t* test; *p* < 0.05, Bonferroni corrected), indicating that γ events tend to appear at specific phases during low-frequency oscillations (Fig. 13*B*).

## Discussion

We studied individual oscillation events in auditory cortex of NHPs and humans in large, invasively-recorded, nontask-related datasets. We found near-continuous (90% of time) neuronal oscillations across the full range of frequency bands. Oscillations occurred in events of up to 44 cycles (Fig. 7*F*), average three to four cycles (Fig. 9*A*). We developed a software package, OEvent, to identify and characterize events from electrophysiological signals using wavelet transform, after removal of ERPs and broadband noise. We validated OEvent using simulated oscillations superimposed on the fluctuating background from NHP recordings (Fig. 3).

OEvent tools demonstrated in the present study provides an open source, easy-to-use, and automated method for researchers to carry out oscillation event detection and characterization in electrophysiological recordings, with the hopes that more researchers would benefit from OEvent to study the dynamics of oscillation events at the single trial level in the context of different cognitive processes. The main strengths and novelty of OEvent lies in the detection procedure itself that applies a local maximum filter to detect peaks in the wavelet spectrograms, and then produces a bounding box around each oscillation event. This enables the calculation of various event features including frequency span, time span, peak frequency, and other customizable features that are not discussed in this study, but might be of interest for particular investigations. In addition to event detection and characterization, we demonstrate the application of these procedures and the use of the different characteristics to explore oscillations at the single trial level or ongoing recordings.

While the initial step of the algorithm relies on the widely used wavelet transform, it is not the only way to represent the signal in the time-frequency domain. Depending on the question at hand, users may opt to use other methods, such as short-time Fourier transform (STFT), as the first step, followed by the detection procedures and features extraction using OEvent.

Of special interest, more recent examples include the superlet package (Moca et al., 2021), a wavelet method that achieves good temporal resolution and frequency resolution by using geometrically-combined sets of wavelets, and the matching pursuit (MP) algorithm (S Chandran et al., 2016) that has the ability to represent sharp transients in the signal by creating a redundant dictionary of tiles of different sizes in the time and frequency domains. Other recent innovations include detection of nonsinusoidal waveform shapes, such as sharp sawtooth waveforms observed during hippocampal θ oscillations (Cole and Voytek, 2017; Donoghue et al., 2022) or frontocentral cortical activity during rapid eye movement sleep (Frauscher et al., 2020). As noted, OEvent could be adapted to detect these more diverse waveform shapes using specially-crafted wavelet filters.

In the present study, since we focused on characterizing oscillation events within different bands, we chose the more widely used wavelet transform with a constant width to achieve more comparable results across bands. Our simulations show that OEvent can reliably detect the number of cycles and peak frequency of oscillation events with high accuracy for most frequency bands, and at multiple event durations. In particular, higher accuracy was achieved by OEvent in detecting the number of cycles of an oscillation event as seen in Figure 3. Since the wavelet approach in OEvent is similar to other methods, the differences may be attributed to the additional steps taken in OEvent, specifically, reducing the contribution of ERP-like events, applying a local maximum filter, and creating the bounding boxes based on an adaptive threshold that is frequency specific.

In the neural data, basic characterization (Fig. 9*D*) showed events that generally remained within a classical frequency-band but were nonconstant (see U-shape in Figs. 5, 6, α), with intraevent shift ∼65% on average. Oscillation events within a particular band were regular (CV2 and FF ≪1), related to consistent cross-frequency coupling. Overall, our results showed that neuronal oscillations across a wide range of frequencies occur in quasi-rhythmic, multicycle events, or “bursts,” with significant across-burst temporal predictability.

### Importance of oscillations

Although the term “resting state” is often used, the awake brain is never functionally at rest (Miall and Robertson, 2006; Zuo et al., 2010). We prefer “nontask-related” to “resting” state, noting that our conditions allow for ongoing perception and thought to produce ever-changing brain activity, and therefore great variation in oscillation events.

Oscillations are sometimes considered to be functionally irrelevant, or epiphenomenological. By contrast, we and others view oscillations as a fundamental reflection of brain function and information encoding. Therefore, study and classification of oscillations will be critical for understanding the brain (Thut et al., 2012). Prior studies suggest rhythmic oscillatory bursting as a dominant mode in auditory cortex that allows encoding of acoustic signals with information at multiple time scales (Rosen, 1992). In one study, γ peak frequencies depended on level of arousal, with amplitude more dependent on attention (Lakatos et al., 2004). Another study found precise and distinct timing of β versus γ oscillations during musical beat processing (Fujioka et al., 2009). Related research in human has also shown event-like β and γ oscillations with auditory processing and auditory-motor interactions (Large, 2000; Snyder and Large, 2005; Fujioka et al., 2012; Abbasi and Gross, 2020). In a mnemonic task, both β and γ were found to contribute to working memory processes (Lundqvist et al., 2016).

From our study, we would suggest that maintenance of temporal coupling across bands in absence of input may provide “readiness” for processing of incoming auditory information. Subsequently, perceptual input during our recordings would then capture and entrain ongoing events as well as creating new ones. We note that PAC between slow and fast oscillations increased during events (Fig. 10), an association consistent with phonemic and syllabic rates in speech to which both our animals and humans were exposed (Arnal et al., 2015a, b).

### Oscillation event variability

Several previous studies have used rigid criteria to identify oscillations, typically identifying an oscillation as a single frequency with relatively constant amplitude across cycles (Cole and Voytek, 2019; Donoghue et al., 2020). We cast our net more widely to capture the enormous variety of oscillatory phenomenology, having identified gradual frequency and power shifts in auditory processing (Giraud and Poeppel, 2012; Teng et al., 2017). Strict classification also obscures the enormous nonstationarity of brain signals from task-independent fluctuations because of perception, cognition and behavioral demands (Musall et al., 2019). Our less restrictive approach identified cycle-to-cycle variations in frequency and amplitude.

Interpretation of rhythms and rhythmic “motifs” relies both on measurement technology and methods of analysis (de Cheveigné and Nelken, 2019). Different recording types produce different signals that require different analyses: extracranial versus intracranial, intracranial depth versus surface, monopolar versus bipolar, LFP versus CSD. Different frequency bands also require different analyses, as do different brain areas, and different task conditions. In the current study, we were encouraged by obtaining similar results across different measurement technologies (iEEG, LFP, CSD, MUA) and across species. Rather than analyzing in only the frequency domain (Voytek et al., 2015) or only the time domain (Neymotin et al., 2008), we have found advantages in moving back and forth across these different views. For example, we could better exclude ERPs using features from both domains: waveform shape and duration (time-domain), and frequency-span (frequency-domain). Our analysis on the quasi-rhythmicity of oscillation events relied on first defining the events using the frequency domain (wavelet transform) and then using time-domain to measure interevent rhythmicity (CV2 of IEIs).

While individuals will disagree on oscillation identification, we found many intuitively clear examples of oscillations with sinusoidal appearance (Figs. 2-5). Additionally, we presented here the signal heterogeneity in our dataset (Fig. 5) to allow the readers to see their variability, and provide confidence that these oscillations are not artifacts of the choice of wavelet filter properties. By sharing the current datasets and the OEvent software package, we hope to encourage further sharing for cross-validation of both tools and datasets. We expect that gradual agreement on consistent sets for oscillation analysis and scoring will help the community come to a consensus on features to distinguish oscillations versus event-driven waveforms.

### Oscillation mechanisms

Biophysical computer modeling of neurons (Neymotin et al., 2017; Kelley et al., 2021) and detailed microcircuits can be used to predict oscillation variability by identifying their origins in cellular properties, and in the interaction of multiple generators (Neymotin et al., 2011a,b; Dura-Bernal et al., 2019; Neymotin et al., 2020; Bonaiuto et al., 2021; Law et al., 2022). The interactions among classes of interneurons, and with pyramidal cells, contribute to fast and slow rhythms, via short and long GABAA synaptic time constants (Skinner et al., 1994; Whittington et al., 2000; Vierling-Claassen et al., 2010; Neymotin et al., 2011a), creating a mix of oscillations with variation in frequencies and amplitude (Cardin et al., 2009; Cardin, 2012; Fukunaga et al., 2014; Chen et al., 2017; Naka et al., 2019). Using a large-scale biophysically and anatomically realistic model, transient γ oscillations were suggested to be intrinsically generated by the basolateral amygdala nucleus (Feng et al., 2019). Feng et al. (2021) also suggested that although different populations of interneurons support β and γ oscillations, they share the same local neocortical microcircuit, with different short-term synaptic plasticity. Modeling could allow us to further extend oscillation taxonomies by distinguishing relationships with circuit elements (Ainsworth et al, 2011; Dura-Bernal et al., 2019, 2022; Park and Geffen, 2020). A clearer understanding of the mechanisms generating oscillations will also pave the way to better understanding of neuropathology associated with disrupted brain rhythms (Sherif et al., 2020), and how circuit features contribute to support particular aspects of auditory processing, such as speech tracking (Ghitza et al., 2008; Shamir et al., 2009; Pittman-Polletta et al., 2021), and auditory steady-state responses (Kudela et al., 2018; Metzner et al., 2019; Lakatos et al., 2020).

### Ubiquity of oscillation events

To our knowledge, ours is the first study to systematically quantify the full range of physiological cortical oscillations rather than focusing on particular bands. Overall our findings agree with prior studies of β in somatosensory (Sherman et al., 2016; Shin et al., 2017), motor (Feingold et al., 2015; Little et al., 2019; Wessel, 2020), and frontal cortex (Lundqvist et al., 2018). However, we found 3.6–3.8 cycles per event, somewhat higher than the <3 cycles found in these prior studies, because of our allowing intraevent frequency shift and to our lower threshold. β Events showed occurrence rates of ∼1.4–1.5 Hz, comparable to previous studies (Sherman et al., 2016; Shin et al., 2017). Turning to γ, we agree here with prior findings of nonstationary and discrete (event-like) γ (Lakatos et al., 2004). As also noted here, PAC has been reliably found in auditory cortex (Lakatos et al., 2005) and other brain areas (Sirota et al., 2008; Tort et al., 2008; Dvorak and Fenton, 2014; O’Connell et al., 2015). Additional recent studies have identified spatial location of γ bursts associated with α traveling over the cortical surface (Bahramisharif et al., 2013).

In conclusion, we find enormous variability in oscillatory brain activity. but with enough consistency to point to involvement of recurrent perceptual or cognitive events that could be further explored in a task-related context. Such study will permit better understanding of the role of neuronal oscillations in information processing and in disease (Kopell et al., 2010; Buzsáki and Watson, 2012; Pittman-Polletta et al., 2015; Javitt et al., 2020).

## Synthesis

Reviewing Editor: Arvind Kumar, KTH Royal Institute of Technology Decisions are customarily a result of the Reviewing Editor and the peer reviewers coming together and discussing their recommendations until a consensus is reached. When revisions are invited, a fact-based synthesis statement explaining their decision and outlining what is needed to prepare a revision will be listed below. The following reviewer(s) agreed to reveal their identity: Benjamin Morillon.

The two reviewers are happy with the revision. Most of their concerns have been addressed. There are a couple of minor issues remain which we believe can be addressed in the production process. Please make sure that the following two minor issues are addressed in the proofreading stage:

Now I only have two minor concerns need to be addressed before its publication:

1. Line 205, please explain what kind of filter was applied when authors mentioned “local maximum filter”?

2. In fig 3B and 3C, are the filtered (blue) signals in two bottom plots from wavelet transform?
