# Supplementary material for: Detecting Spontaneous Neural Oscillation Events in Primate Auditory Cortex
Source: eNeuro. 2022 Aug 18;9(4):ENEURO.0281-21.2022. doi: 10.1523/ENEURO.0281-21.2022 (PMC9395248; doi:10.1523/ENEURO.0281-21.2022)
Supplement: Extended Data Table 8-1 — Average event rate (Hz) ± SEM, and event count in parentheses, for the different physiological oscillation frequency bands. A1 Supra, A1 Gran, and A1 Infra are from NHP A1 supragranular, granular, and infragranular sink channels, respectively. STG is from human iEEG recorded in supratemporal gyrus. Download Table 8-1, DOCX file. [file enu-eN-NWR-0281-21-s03.docx]

| **Event Rate** | **Delta** | **Theta** | **Alpha** | **Beta** | **Low Gamma** | **Gamma** | **High Gamma** |
| --- | --- | --- | --- | --- | --- | --- | --- |
| **A1 Supra** | 0.36+/-0.0 (10,600) | 0.56+/-0.0 (16,544) | 0.66+/-0.01 (19,476) | 1.54+/-0.01 (44,920) | 1.29 +/-0.01 (37,800) | 4.44+/-0.01 (130,088) | 13.12+/-0.02 (383,848) |
| **A1 Gran** | 0.36+/-0.0 (10,519) | 0.58+/-0.0 (17,028) | 0.68+/-0.0 (20,032) | 1.51+/-0.01 (44,266) | 1.27 +/-0.01 (37,089) | 4.41+/-0.01 (129,129) | 13.08+/-0.02 (382,922) |
| **A1 Infra** | 0.36+/-0.0 (10,563) | 0.57+/-0.0 (16,756) | 0.64+/-0.00 (18,874) | 1.43+/-0.01 (41,912) | 1.23 +/- 0.01 (35,972) | 4.41+/-0.01 (129,075) | 13.18+/-0.02 (385,638) |
| **STG** | 0.37+/-0.01 (820) | 0.55+/-0.01 (1,204) | 0.74+/-0.02 (1,630) | 1.50+/-0.02 (3,287) | 1.28 +/- 0.02 (2819) | 3.29+/-0.05 (7,246) | 12.26+/-0.07 (26,917) |

**Table 8-1. Average Event Rate (Hz) +/- standard error of the mean, and event count in parentheses, for the different physiological oscillation frequency bands.** A1 Supra, A1 Gran, A1 Infra are from NHP A1 supragranular, granular, and infragranular sink channels, respectively. STG is from human iEEG recorded in supratemporal gyrus.
