# Supplementary material for: Detecting Spontaneous Neural Oscillation Events in Primate Auditory Cortex
Source: eNeuro. 2022 Aug 18;9(4):ENEURO.0281-21.2022. doi: 10.1523/ENEURO.0281-21.2022 (PMC9395248; doi:10.1523/ENEURO.0281-21.2022)
Supplement: Extended Data Table 8-2 — ATR for the different physiological oscillation frequency bands. Mean value is presented (SE was negligible). A1 Supra, A1 Gran, and A1 Infra are from NHP A1 supragranular, granular, and infragranular sink channels, respectively. STG is from human iEEG recorded in supratemporal gyrus. Download Table 8-2, DOCX file. [file enu-eN-NWR-0281-21-s04.docx]

| **ATR** | **Delta** | **Theta** | **Alpha** | **Beta** | **Low Gamma** | **Gamma** | **High Gamma** |
| --- | --- | --- | --- | --- | --- | --- | --- |
| **A1 Supra** | 0.44 | 0.29 | 0.19 | 0.25 | 0.13 | 0.26 | 0.34 |
| **A1 Gran** | 0.43 | 0.29 | 0.20 | 0.25 | 0.13 | 0.26 | 0.34 |
| **A1 Infra** | 0.44 | 0.30 | 0.19 | 0.23 | 0.13 | 0.25 | 0.34 |
| **STG** | 0.46 | 0.30 | 0.21 | 0.25 | 0.13 | 0.20 | 0.32 |

**Table 8-2. Active time ratio (ATR) for the different physiological oscillation frequency bands.** Mean value is presented (standard error was negligible). A1 Supra, A1 Gran, A1 Infra are from NHP A1 supragranular, granular, and infragranular sink channels, respectively. STG is from human iEEG recorded in supratemporal gyrus.
