# Supplementary material for: Detecting Spontaneous Neural Oscillation Events in Primate Auditory Cortex
Source: eNeuro. 2022 Aug 18;9(4):ENEURO.0281-21.2022. doi: 10.1523/ENEURO.0281-21.2022 (PMC9395248; doi:10.1523/ENEURO.0281-21.2022)
Supplement: Extended Data Table 9-1 — Cycles Per Event for the different physiological oscillation frequency bands. Range and mean ± SEM, separated by semicolon (;). A1 Supra, A1 Gran, and A1 Infra are from NHP A1 supragranular, granular, and infragranular sink channels, respectively. STG is human iEEG signals recorded from supratemporal gyrus. Download Table 9-1, DOCX file. [file enu-eN-NWR-0281-21-s05.docx]

**Tables 9-1** through **9-4** list ranges and mean+/-standard error of the mean.

| **Cycles Per Event** | **Delta** | **Theta** | **Alpha** | **Beta** | **Low Gamma** | **Gamma** | **High Gamma** |
| --- | --- | --- | --- | --- | --- | --- | --- |
| **A1 Supra** | 0.4-12.4; 2.8+/-0.0 | 0.7-17.2; 3.3+/-0.0 | 1.1-21.1; 3.5+/-0.0 | 1.1-23.5; 3.6+/-0.0 | 1.0-20.1; 3.6+/-0.0 | 0.7-30.4; 3.7+/-0.0 | 0.6-37.8; 3.8+/-0.0 |
| **A1 Gran** | 0.5-11.1; 2.8+/-0.0 | 0.7-18.9; 3.3+/-0.0 | 0.7-19.6; 3.5+/-0.0 | 0.8-31.4; 3.7+/-0.0 | 0.6-25.6; 3.66+/-0.0 | 0.6-30.0; 3.7+/-0.0 | 0.5-44.2; 3.8+/-0.0 |
| **A1 Infra** | 0.3-13.1; 2.8+/-0.0 | 0.9-18.6; 3.4+/-0.0 | 0.7-27.9; 3.5+/-0.0 | 0.6-21.8; 3.6+/-0.0 | 0.6-26.7; 3.6+/-0.0 | 0.5-30.0; 3.6+/-0.0 | 0.5-34.9; 3.7+/-0.0 |
| **STG** | 0.7-11.8; 3.0+/-0.0 | 1.3-14.7; 3.7+/-0.1 | 1.0-25.9; 3.5+/-0.0 | 1.3-26.4; 3.8+/-0.0 | 1.3-25.2; 3.73+/-0.0 | 0.7-32.4; 3.24+/-0.0 | 0.7-38.8; 3.49+/-0.0 |

**Table 9-1. Cycles Per Event for the different physiological oscillation frequency bands.** Range and mean+/-standard error of the mean, separated by semicolon (;). A1 Supra, A1 Gran, A1 Infra are from NHP A1 supragranular, granular, and infragranular sink channels, respectively. STG is human iEEG signals recorded from supratemporal gyrus.
