# Supplementary material for: Detecting Spontaneous Neural Oscillation Events in Primate Auditory Cortex
Source: eNeuro. 2022 Aug 18;9(4):ENEURO.0281-21.2022. doi: 10.1523/ENEURO.0281-21.2022 (PMC9395248; doi:10.1523/ENEURO.0281-21.2022)
Supplement: Extended Data Table 9-2 — Number of local peaks in filtered waveforms for the different physiological oscillation frequency bands. Range and mean ± SEM are presented, separated by semicolon (;). A1 Supra, A1 Gran, A1 Infra are from NHP A1 supragranular, granular, and infragranular sink channels, respectively. STG is human iEEG signals recorded from supratemporal gyrus. Download Table 9-2, DOCX file. [file enu-eN-NWR-0281-21-s06.docx]

| **Local Peaks** | **Delta** | **Theta** | **Alpha** | **Beta** | **Low Gamma** | **Gamma** | **High Gamma** |
| --- | --- | --- | --- | --- | --- | --- | --- |
| **A1 Supra** | 0-13; 3.1+/-0.0 | 1-19 3.5+/-0.0 | 1-22; 3.6+/-0.0 | 0-28; 3.7+/-0.0 | 0-30; 3.7+/-0.0 | 0-42; 3.8+/-0.0 | 0-36; 3.8+/-0.0 |
| **A1 Gran** | 0-16; 3.2+/-0.0 | 1-26; 3.5+/-0.0 | 0-26; 3.6+/-0.0 | 0-30; 3.7+/-0.0 | 0-26; 3.7+/-0.0 | 0-32; 3.8+/-0.0 | 0-40; 3.8+/-0.0 |
| **A1 Infra** | 0-16; 3.2+/-0.0 | 0-26; 3.5+/-0.0 | 0-22; 3.6+/-0.0 | 0-31; 3.7+/-0.0 | 0-32; 3.7+/-0.0 | 0-46; 3.7+/-0.0 | 0-35; 3.8+/-0.0 |
| **STG** | 1-15; 3.2+/-0.1 | 1-21; 3.9+/-0.1 | 1-19; 3.6+/-0.0 | 1-25; 3.8+/-0.1 | 1-16; 3.5+/-0.0 | 1-26; 3.1+/-0.0 | 0-33; 3.4+/-0.0 |

**Table 9-2. Number of local peaks in filtered waveforms for the different physiological oscillation frequency bands.** Range and mean+/-standard error of the mean are presented, separated by semicolon (;). A1 Supra, A1 Gran, A1 Infra are from NHP A1 supragranular, granular, and infragranular sink channels, respectively. STG is human iEEG signals recorded from supratemporal gyrus.
