# Supplementary material for: Detecting Spontaneous Neural Oscillation Events in Primate Auditory Cortex
Source: eNeuro. 2022 Aug 18;9(4):ENEURO.0281-21.2022. doi: 10.1523/ENEURO.0281-21.2022 (PMC9395248; doi:10.1523/ENEURO.0281-21.2022)
Supplement: Extended Data Table 9-3 — Logarithmic frequency bandwidth (Fspan) for the different physiological oscillation frequency bands. Values are mean ± SEM. A1 Supra, A1 Gran, and A1 Infra are from NHP A1 supragranular, granular, and infragranular sink channels, respectively. STG is human iEEG signals recorded from supratemporal gyrus. Download Table 9-3, DOCX file. [file enu-eN-NWR-0281-21-s07.docx]

| **Fspan** | **Delta** | **Theta** | **Alpha** | **Beta** | **Low Gamma** | **Gamma** | **High Gamma** |
| --- | --- | --- | --- | --- | --- | --- | --- |
| **A1 Supra** | 0.580+/-0.002 | 0.500+/-0.002 | 0.490+/-0.002 | 0.490+/-0.001 | 0.480+/-0.001 | 0.480+/-0.001 | 0.470+/-0.000 |
| **A1 Gran** | 0.580+/-0.002 | 0.490+0.002 | 0.480+/-0.002 | 0.480+/-0.001 | 0.480+/-0.001 | 0.480+/-0.001 | 0.470+/-0.000 |
| **A1 Infra** | 0.590+/-0.002 | 0.500+/-0.002 | 0.490+/-0.002 | 0.480+/-0.001 | 0.480+/-0.001 | 0.470+/-0.001 | 0.470+/-0.000 |
| **STG** | 0.600+/-0.009 | 0.530+/-0.007 | 0.500+/-0.006 | 0.530+/-0.004 | 0.490+/-0.004 | 0.430+/-0.003 | 0.470+/-0.001 |

**Table 9-3. Logarithmic frequency bandwidth (Fspan) for the different physiological oscillation frequency bands.** Values are mean+/-standard error of the mean. A1 Supra, A1 Gran, A1 Infra are from NHP A1 supragranular, granular, and infragranular sink channels, respectively. STG is human iEEG signals recorded from supratemporal gyrus.
